# Development of novel reinforcement learning-based optimizer to impede tumor growth via radiochemotherapy

**DOI:** 10.1038/s41598-026-56355-2

**Published:** 2026-06-16

**Authors:** Muhammad Arsalan, Xiaojun Yu, Muhammad Tariq Sadiq

**Affiliations:** 1 School of Automation, Northwestern Polytechnical University, Xi’an, 710072 China; 2 School of Computer Science and Electronic Engineering, University of Essex, Colchester, CO4 3SQ UK

**Keywords:** Radiotherapy, Chemotherapy, Nonlinear sliding mode control (SMC), Tumor mitigation, Proximal policy optimization (PPO), Cancer, Computational biology and bioinformatics, Mathematics and computing, Oncology

## Abstract

Current cancer treatment strategies prioritize algorithmic robustness and efficiency but frequently neglect critical aspects of patient safety and comfort. These approaches typically rely on chemotherapy-based mathematical models optimized solely for short-term tumor reduction, disregarding the broader impact on patient health. This study introduces a patient-centered approach to optimize cancer treatment, balancing treatment efficacy and toxicity. The proposed research method incorporates both radiation therapy and chemotherapy simultaneously in the form of ordinary differential equations (ODE)-based mathematical dynamics. These updated dynamics are then utilized to propose a novel control mechanism that integrates nonlinear sliding mode control (SMC) with reinforcement learning-based proximal policy optimization (PPO) algorithm. Conventional sliding mode control (SMC) algorithm is first modified by replacing its signum function-based switching control with a sigmoid function to address issues like chattering and transients in treatment control. This smooth SMC is then incorporated within the framework of PPO to dynamically adjust treatment schedules, reduce drug and radiation dosages, smooth administration of treatment dosages, and enhance patient health indicators. Results showed that the proposed hybrid PPO method effectively lowered chemotherapy and radiotherapy dosages while maintaining tumor suppression, minimizing treatment toxicity, and improving immune cell recovery. In quantitative comparisons, the proposed PPO algorithm reduced baseline dosages by up to 76.8% for chemotherapy and 66% for radiotherapy and achieved tumor suppression 5.67% faster than conventional multi-input optimization methods. It also lowered cumulative treatment intensity by over 92%, demonstrating a substantial enhancement in patient safety. The methodological originality of this study lies in integrating nonlinear smooth SMC with reinforcement learning-based PPO within a patient-centered ODE modeling framework that jointly represents radiotherapy, chemotherapy, tumor dynamics, immune cell dynamics, healthy cell preservation, and health indicator state. The proposed framework provides a relevant, toxicity-aware computational approach for radio-chemotherapy dosage optimization, demonstrating lower simulated treatment intensity while maintaining tumor suppression under stated model assumptions. As a pre-clinical computational proof of concept, this work establishes a robust and interpretable basis for future treatment-planning studies, subject to retrospective clinical validation, patient-specific parameterization, and prospective safety evaluation.

## Introduction

A tumor refers to the formation of an abnormal mass of cells that arises due to uncontrolled cell proliferation and may be benign or malignant in nature^1^. Cancer, in contrast, is characterized by malignant tumors that possess the ability to invade surrounding tissues, evade immune surveillance, and metastasize to distant organs, making it one of the leading causes of morbidity and mortality worldwide^2^. The number of confirmed cases of cancer are expected to rise to 35 million by 2050 from 20 million in 2022^3,4^. Moreover, around 10 million people lose their lives annually due to this lethal condition^3^. Early diagnosis and immediate therapy initiation are key for survival against this complex and fatal disease^5^. Current treatment options include radiotherapy, chemotherapy, immunotherapy, hormone therapy, antiangiogenic therapy, targeted therapy, monoclonal antibody therapy, and tissue removal through surgery^6–9^. The latest studies emphasize the utilization of combinations of two or more of these therapies and treatment plans to combat the menace of cancer more effectively^9,10^.

Clinically, according to the Stupp protocol, removal of tumorous tissue through surgery, followed by concurrent radiation therapy and temozolomide (TMZ)-based chemotherapy is considered as the primary first-line treatment^11,12^. The Stupp protocol refers to the standard treatment regimen for glioblastoma, consisting of surgical resection followed by concurrent radiotherapy and TMZ chemotherapy, and subsequent adjuvant TMZ administration, which has been shown to significantly improve survival^11,12^. It should be noted that TMZ-based chemotherapy is not a universal first-line treatment for all cancer types; rather, it is primarily employed in the management of malignant gliomas, particularly glioblastoma, due to its ability to cross the blood-brain barrier and its established clinical efficacy in the context. However, the administration of combined radio-chemotherapy after surgical resection is still a standard protocol, applied to manage and treat most of the cancer advancements^11^. If this initial and preferred approach fails due to the developed resistance by tumor tissue or the side effects of employed therapy becomes intolerable, then second-line alternate therapies are utilized. These procedures utilize growth inhibitors along with simultaneous administration of chemotherapy and radiation to curb the advancements of tumor^13,14^. Apart from growth inhibitors, immunotherapy or some other novel targeted therapies may also be applied if the tumor progression remains unaffected^15^. Another crude but prevalent procedure involves administration of treatment dosages in linear pattern at fixed intervals. Occasionally, clinical evaluation of tumor progression using magnetic resonance imaging (MRI) after every two or three months is also employed to alter the regimen dosages based on disease advancements^16^. However, all the procedures and drugs employed by these treatment plans have adverse side effects^11,12^. Thus, novel strategies to optimize the drug and radiation dosages are needed so that overdosing may be avoided and that cancerous and tumor cells may be purged, while ensuring that human health is not compromised^17,18^.

Experimental work in the area of tumor-subjugation is not only substantially costly, but also involves time- and resource-intensive trials^19^. Thus, extensive work has been carried out to develop tumor-immune system dynamics and their control methodologies^20,21^. Recent studies have proposed numerous techniques and algorithms based on ordinary differential equations (ODE)-based tumor growth models to analyze and contain tumor spread by employing unique treatment plans. These models describe the temporal evolution of tumor, immune, and healthy cell populations using deterministic mathematical formulations, enabling the analysis of tumor progression and therapeutic interventions under controlled assumptions. Classical tumor growth formulations, such as the Gompertz model, are examples of ODE-based models, and have been widely used to describe tumor proliferation dynamics under limited resource conditions^22^. Similarly, Wu et al.^22^ and Sena et al.^23^ proposed a three state model that includes tumor dynamics as well as drug interaction with tumor tissue, and its toxicity in the blood stream. In actuality, tumor progression stimulates the immune system of the human body and thus results in a dynamical interaction between tumorous and immune cells during anticancer therapy. Thus, Shindi et al.^20^, Sarhaddi et al.^24^ and Qaiser et al.^2^ not only included the dynamics of immune system, but also modeled the impact of chemotherapeutic drugs on them. Kumar et al.^10^ further modified the three state basic ODE model to a five state system that incorporates both radiation and chemotherapeutic drugs as control inputs for tumor reduction. However, none of these recent studies have considered the impact of radiation or chemotherapy on the actual health and well-being of patients^25,26^.

Recently, numerous nonlinear control algorithms have been proposed to contain the advancement of cancerous tumors^27–29^. These algorithms are designed to regulate systems whose dynamics exhibit strong non-linearities, parameter uncertainties, and complex interactions among state variables^24^. Since the tumor-immune system dynamics are inherently nonlinear due to feedback interactions, growth saturation, and treatment-induced perturbations, nonlinear controllers are naturally more suitable and effective for tumor mitigation compared to linear control approaches^2,10^. Fractional order tumor models extend conventional integer-order differential equation models by incorporating fractional-order derivatives, allowing the representation of memory and hereditary effects in tumor growth dynamics^28^. Combined adaptive fuzzy SMC algorithm integrates fuzzy logic-based decision rules with the robustness of SMC, enabling adaptive adjustment of control actions in the presence of system uncertainties and non-linearities^24^. A fractional-order tumor model is utilized to design a combined adaptive fuzzy and sliding mode controller to exploit the benefits of both algorithms^24^. Similarly, finite-time fuzzy controllers are designed to converge system states to desired equilibrium point within finite time by utilizing fuzzy logic rule-base that adapt control actions based on system behavior. A finite-time fuzzy controller was designed to adjust chemo-dosage for tumor treatment^28^. Nonlinear state-feedback controllers regulate system behavior by directly incorporating nonlinear functions representing system states into the control law^2^. Synergetic controllers enforce pre-defined invariant manifolds to ensure stable convergence of system trajectories^30^. Whereas, fuzzy logic controllers employ rule-based reasoning to handle uncertainties and converge system states to desired outcomes^28^. Qaiser et al.^2^ studied and compared the effectiveness of nonlinear state feedback, synergetic and fuzzy logic controllers for drug administration in tumor-affected patients. Integral SMC and double integral SMC are two variants of SMC devised to mitigate any steady state error that may arise due to system parameter mismatch or some external disturbances. Similarly, by replacing the equivalent control of SMC with a super-twisting algorithm based control law while incorporating the switching term of SMC, a hybrid super-twisting SMC algorithm is devised. Zubair et al.^31^ designed and compared different variants of sliding mode control including integral, double-integral and super-twisting SMCs for the chemotherapy of brain tumors. Jiao et al.^32^ proposed a two-state tumor-immune system interaction model for the development of a Lyapunov-based adaptive controller to inhibit tumor growth. These algorithms utilize Lyapunov stability theory to devise an adaptive control strategy to dynamically adjust control parameters in the presence of uncertainties. Moreover, the model considered tumor cell and immune cell dynamics as coupled state variables, capturing the complex interaction between tumor proliferation and immune-mediated tumor suppression. Similarly, smooth nonlinear super-twisting and synergetic control algorithms aim to achieve finite-time convergence while reducing chattering by employing continuous control laws and invariant manifold-based stabilization^30^. Rsetam et al.^29^ and Arsalan et al.^30^ proposed smooth nonlinear supertwisting and synergetic control algorithms respectively to mitigate cancerous tumor.

In addition to nonlinear dynamics, biomedical systems have numerous critical constraints and limits, which need to be observed and managed. Optimal control theory focuses on determining control inputs that minimize or maximize a predefined objective function subject to system dynamics and constraints, and has been widely applied to optimize treatment schedules in tumor therapy^33,34^. The optimal control theory has been combined with the effectiveness of multi-objective particle swarm optimization (PSO) to determine the optimized chemo dosage for treating cancerous tumor^20^. PSO is a population-based metaheuristic inspired by the collective behavior of social organisms, where candidate solutions iteratively evolve toward optimal regions of the search space. Multi-objective PSO extends this framework to simultaneously optimize multiple conflicting objectives. Hybrid controller composed of PSO optimized fuzzy logic controller and classic proportional-derivative (PD) technique has been employed to determine the chemotherapeutic drug dosage^23^. A hybrid controller combines two or more control paradigms, such as model-based nonlinear and data-driven approaches, to leverage their complementary strengths for improved performance and robustness. The PD technique regulates system behavior by applying corrective actions proportional to the error and its rate of change, offering simplicity but limited robustness for highly nonlinear systems. Similarly, Kalman filter based optimal controller and switched system optimal controller were proposed for tumor reduction in Mohite et al.^35^, Belkhatir et al.^36^ and Wu et al.^22^. Kalman filter-based optimal controllers incorporate state estimation to optimally regulate systems affected by noise and uncertainty, while switched optimal controllers employ multiple control laws that are activated based on predefined system conditions. The finite-set model predictive control (MPC) and nonlinear MPC were proposed for anti-tumor therapies by Javadi et al.^37^ and Villa et al.^38^. Finite-set MPC evaluates a finite number of candidate control actions to select an optimal input, while nonlinear MPC extends predictive control frameworks to explicitly account for nonlinear system dynamics and constraints. Kumar et al.^10^ proposed a linear optimal controller for multi-input tumor-immune system model to regulate radiation as well as chemotherapeutic dosages and have shown that a combined therapy is more potent and effective against cancerous tumors. The linear optimal controller determines treatment inputs by optimizing a linearized representation of the system dynamics under multiple therapeutic interventions. Similarly, high dosages of chemotherapeutic drugs pose a number of health related challenges, thus Arora et al.^39^ proposed metronomic chemotherapy to study the impact of low but frequent dosages of chemotherapeutic drugs on tumor proliferation. Metronomic chemotherapy refers to the administration of low-dose chemotherapeutic drugs at regular and frequent intervals to minimize toxicity while maintaining therapeutic efficacy.

Reinforcement learning (RL) based algorithms are specialized machine learning (ML) techniques that train an agent through a mechanism to award penalty or reward against any performed action^40^. The agent then explores among the possible optimized strategies on the basis of learned penalties and rewards. Thus, by doing so, the desired objective can be achieved. Hence, RL can be employed effectively to solve optimization problems. RL-based approaches have been proposed to control the amount of radiation dosage for the purpose of tumor shrinkage^41–43^. Similarly, RL algorithms have been recently employed to manage chemotherapeutic drug dosage to kill cancerous tumor cells^44–46^. The RL based algorithm has also been applied to limit tumor growth under anti-angiogenic therapy^6^. Whereas, Mashayekhi et al.^7^ proposed deep RL based twin delayed deep deterministic (TD3) policy gradient method to determine the drug dosage of chemotherapeutic drug for cancerous tumor mitigation. The TD3 method is a deep learning algorithm that improves training stability and performance by reducing overestimation bias through delayed policy updates and twin critic networks.

This study addresses a relevant methodological gap in toxicity-aware treatment scheduling by proposing an updated ODE-based nonlinear tumor-immune system interaction model that jointly considers radiotherapy and chemotherapy as control inputs while explicitly representing their impact on a general health indicator state. The model describes the coupled evolution of tumor, immune, and healthy cell populations using nonlinear differential equations, enabling transparent analysis of therapeutic effects and biological interactions over time, within a reproducible computational framework. The central methodological contribution is an original hybrid smooth sliding mode control - proximal policy optimization (SSMC-PPO) algorithm for simultaneous regulation of radiation and chemotherapeutic dosages for tumor mitigation. By combining the robustness of sigmoid function-based smooth SMC with the adaptive optimization capacity of RL algorithm, the proposed controller provides a multi-objective strategy for tumor suppression, dose minimization, smooth treatment scheduling and health-indicator preservation. The proposed hybrid SSMC-PPO algorithm is thus designed to administer anti-tumor treatment dosages smoothly, to avoid the occurrence of any harmful and unwanted transients, without compromising the robustness and efficiency of SMC algorithm. Similarly, the RL component of the proposed algorithm will not only ensure the minimization of tumor cell population, but will also converge the treatment dosages to zero after successful mitigation of tumor. Additionally, these goals will be achieved while administering the optimized dosages of radiation and chemotherapeutic drug, to limit the toxicity of the treatment procedure, by employing a multi-objective cost function. Moreover, the proposed algorithm will have the capability to manage and accommodate numerous biological and physical constraints to establish its practicability in different modes and patient conditions. Lastly, the study also presents a well-detailed comparative analysis between the novel hybrid SSMC-PPO algorithm and techniques proposed in recent times. Within this pre-clinical computational framework, the primary objectives of this study are:

1. Effective tumor decimation: to employ a well coordinated, multi-input multi-objective approach for efficient and robust mitigation of tumor cell population.
2. Toxicity minimization: minimization of the toxic side-effects of anti-tumor treatment therapies through a multi-objective approach by smart regulation and management of treatment dosages.
3. Enhanced therapeutic effectiveness: minimization of overall intensity of collective treatment dosages of administered radiation and chemotherapeutic drug while efficiently adhering to comply with all the therapeutic considerations.

The study thus aims to robustly halt tumor progression, preserve healthy cell populations, and regulate radiation and chemotherapeutic drug dosages to minimize toxicity and adverse transients. By meeting these objective criteria, tumor subjugation and toxicity minimization can be realized while maintaining or even improving the overall health conditions of the patient under consideration with minimum risks and maximum safety. While the proposed control framework is general and can be extended to other tumor types, this study specifically investigates a brain tumor case study due to its clinical relevance and treatment complexity.

## Materials and methods

This research utilizes a transparent mathematical and simulation-based framework to analyze brain tumor proliferation and the simultaneous effects of radiotherapy and chemotherapy. The adopted methods combine nonlinear control theory with reinforcement learning to evaluate a reproducible computational strategy for tumor mitigation, dose minimization, and health indicator preservation. The study is therefore positioned as a pre-clinical computational proof of concept, rather than a clinically validated treatment protocol.

### Mathematical framework

This study utilizes an ODE-based mathematical model that describes the dynamics of tumor proliferation, intricate interaction of tumor cells with healthy cells, human body’s natural immune response to tumor presence, and the action of considered treatment procedures for tumor mitigation. This research study not only considers the dynamics of both radiotherapy and chemotherapy, but also quantifies the negative impact of the combined treatment procedure in the form of a separate health indicator state. The considered updated model employs the following system state variables:

- T (Tumor cells): represents cancerous brain cell population that proliferates chaotically.
- N (Healthy cells): represents normal brain cell population having direct negative impact of treatment.
- I (Immune cells): represents the response of immune system to the presence of tumor cells.
- H (Health Indicator): represents the deterioration in health due to the combined radio-chemotherapy.
- U (Radiation concentration): represents the amount of radiation accumulated within the body during therapy.
- P (Chemotherapy drug concentration): represents the amount of administered chemotherapeutic drug within body.

The nonlinear tumor model considers two key control inputs, which are regulated by the proposed algorithm to curb tumor proliferation:

1. Radiation dosage (*α*): the radiation dosage administered for tumor mitigation.
2. Chemotherapy dosage (*q*): the administered chemotherapeutic drug dosage for tumor inhibition.

The ODE-based model, describing the complex nonlinear relations among these state variables, and the combined effect of radiation and chemotherapy, as control inputs, provides the foundation for the design of proposed algorithm. Thus, by leveraging this extensive and complex mathematical framework, the multifaceted objective of tumor mitigation, health sustenance and toxicity minimization can be achieved.

### Incorporated dynamics

The following dynamics are considered to provide a comprehensive understanding of the system:

- Tumor and normal cell population: the considered system model utilizes the dynamics of both healthy and tumor cells. Their dynamics not just reflect the influence of therapeutic interventions, but also exhibit the intricate inter-dependencies that characterize their interaction with each other.
- Combined radiotherapy and chemotherapy: the proposed model not only considers the simultaneous impact of radiotherapy and chemotherapy for tumor subjugation, but also includes the detrimental effects of these procedures on healthy and immune cell population.
- Immune system dynamics: to effectively destroy tumor cells, the model incorporates the human immune system’s natural response against tumor proliferation.
- Toxicity minimization and its impact on health: the model estimates the negative impact of treatment therapies on health and personal well-being with the aim to reduce overall toxicity by regulating the administered radiation and chemo drug dosages.

### System parameters

The tumor dynamics employs normalized system parameters. These constants and variables are crucial to simulate practical tumor system dynamics precisely. Key model parameters include:

- Growth rates:
  1. $r_1$: intrinsic growth rate of tumor cells.
  2. $r_2$: growth rate of healthy cells.
  3. $r_3$: recruitment rate of immune cells.
- Killing rates:
  1. $a_{12}$: rate at which healthy cells eliminate tumor cells.
  2. $a_{13}$: rate at which immune cells destroy tumor cells.
  3. $a_{21}$: rate at which tumor cells damage healthy cells.
  4. $a_{31}$: rate at which tumor cells weaken immune cells.
- Carrying Capacities:
  1. $k_{1}$: carrying capacity of tumor cells.
  2. $k_{2}$: carrying capacity of healthy cells.
  3. $k_{3}$: steepness coefficient of tumor specific immune cells.
- Drug and radiation impacts:
  1. $N_{T}$: proportion of tumor cells eliminated by chemotherapy.
  2. $N_{N}$: proportion of healthy cells affected by chemotherapy.
  3. $N_{I}$: proportion of immune cells affected by chemotherapy.
  4. *ε*: fraction of healthy cells affected by radiation.
- Decay rates:
  1. *γ*: decay rate of radiation dosage.
  2. *σ*: decay rate of the chemotherapy drug dosage.

The behavior and dynamics of the proposed system are guided by these parameters and they ensure realistic interactions of the disease and treatment procedure.

### Design goals

The primary design goals of this study are:

1. Effective tumor decimation: to employ a well coordinated, multi-input multi-objective approach for efficient and robust mitigation of tumor cell population.
2. Toxicity minimization: minimization of the toxic side-effects of anti-tumor treatment therapies through a multi-objective approach by smart regulation and management of treatment dosages.
3. Enhanced therapeutic effectiveness: minimization of overall intensity of collective treatment dosages of administered radiation and chemotherapeutic drug while efficiently adhering to comply with all the therapeutic considerations.

By meeting these design and performance criteria, tumor subjugation and toxicity minimization can be realized while maintaining or even improving the overall health conditions of the patient under consideration with minimum risks and maximum safety.

### Dynamical nonlinear model

Most of the previous studies for tumor reduction considered only one control input, i.e. either chemotherapeutic drug or radiation dosage for tumor reduction. Moreover, none of these studies consider any health related consequences that generally arise as a side effect of tumor treatment. The updated model of tumor-immune system interaction, presented here in Eq. (1), considers both chemo and radiation dosages as control inputs. This study is also an initiative to incorporate the harmful impact of these considered treatments on human health, in the form of a separate state, in the mathematical model.

1 $$\begin{aligned} \left\{ \begin{aligned} \dot{T}&= r_1T(1-\frac{T}{k_1}) - a_{12}NT - a_{13}TI - N_T(1-e^{-P})T - UT\\ \dot{N}&= r_2N(1-\frac{N}{k_2}) - a_{21}NT - N_N(1-e^{-P})N - \epsilon UN \\ \dot{I}&= \frac{r_3IT}{T+k_3} - a_{31}IT - d_3I - N_I(1-e^{-P})I \\ \dot{H}&= -{\beta }_1P - {\beta }_2U - {\beta }_3T + {\beta }_4N \\ \dot{U}&= -\gamma U + \alpha \\ \dot{P}&= -\sigma P + q \\ \end{aligned} \right. \end{aligned}$$

The normalized parameter constants used in Eq. (1), along with the units of measurement and brief explanations are presented in Table (1). The values of these constants are considered by exploring and consulting numerous recent scholarly articles^2,10,23,28,39^. The parameters $k_1$ and $k_2$ represent the carrying capacities of tumor and healthy cells respectively, and therefore have units of cells, ensuring dimensional consistency in the logistic growth terms. The parameter $k_3$ represents the steepness coefficient in the tumor-immune interaction terms having units of cells, ensuring the immune recruitment dynamics term to be dimensionally correct^10^. In Eq. (1), “T”, “N”, and “I” represent tumor cells, healthy cells and immune cells respectively. “H” represents health indicator, which is affected by the impact of radiation and chemotherapy. Finally, “U” represents radiation and “P” represents the amount of chemotherapeutic drug inside the body. *α* and *'q'* are the radiation and chemotherapeutic drug control inputs respectively.

**Table 1:** Normalized parameters of dynamical tumor—immune system nonlinear model.

Parameter	Units & Value
$r_1$: Intrinsic growth rate of T	1.5 day$^{-1}$
$r_2$:Intrinsic growth rate of N	1 day$^{-1}$
$r_3$: Recruitment rate of I	0.01 day$^{-1}$
$a_{12}$: Rate of killing of T by N	1 cells$^{-1}\textrm{day}^{-1}$
$a_{13}$: Rate of killing of T by I	0.5 cells$^{-1}\textrm{day}^{-1}$
$a_{21}$: Rate of killing of N by T	1 cells$^{-1}\textrm{day}^{-1}$
$a_{31}$: Rate of killing of I by T	1 cells$^{-1}\textrm{day}^{-1}$
$N_T$: Proportion of T eliminated by chemo	0.5 day$^{-1}$
$N_N$: Proportion of N eliminated by chemo	0.1 day$^{-1}$
$N_I$: Proportion of I eliminated by chemo	0.2 day$^{-1}$
*ε*: fraction of N killed by radiation	0.0008
$d_3$: Natural death rate of I	0.2
$\beta _{1}$: Impact of chemo on BMI	0.2 kg $\textrm{m}^{-2}$ $\textrm{day}^{-1}$
$\beta _{2}$: Impact of radiation on BMI	0.1 kg $\textrm{m}^{-2}$ $\textrm{day}^{-1}$ $\textrm{Gy}^{-1}$
$\beta _{3}$: Impact of T on BMI	0.001 kg $\textrm{m}^{-2}$ $\textrm{day}^{-1}$ $\textrm{cells}^{-1}$
$\beta _{4}$: Impact of N on BMI	0.001 kg $\textrm{m}^{-2}$ $\textrm{day}^{-1}$ $\textrm{cells}^{-1}$
$k_1$: Carrying capacity of T	1 cells
$k_2$: Carrying capacity of N	1 cells
$k_3$: Steepness coefficient of tumor specific immune cells	0.3 cells
*γ*: Decaying rate of radiation	0.082 $\textrm{day}^{-1}$
*σ*: Decaying rate of chemo drug	0.9 $\textrm{day}^{-1}$

### Proposed control methodology

**Fig. 1:**
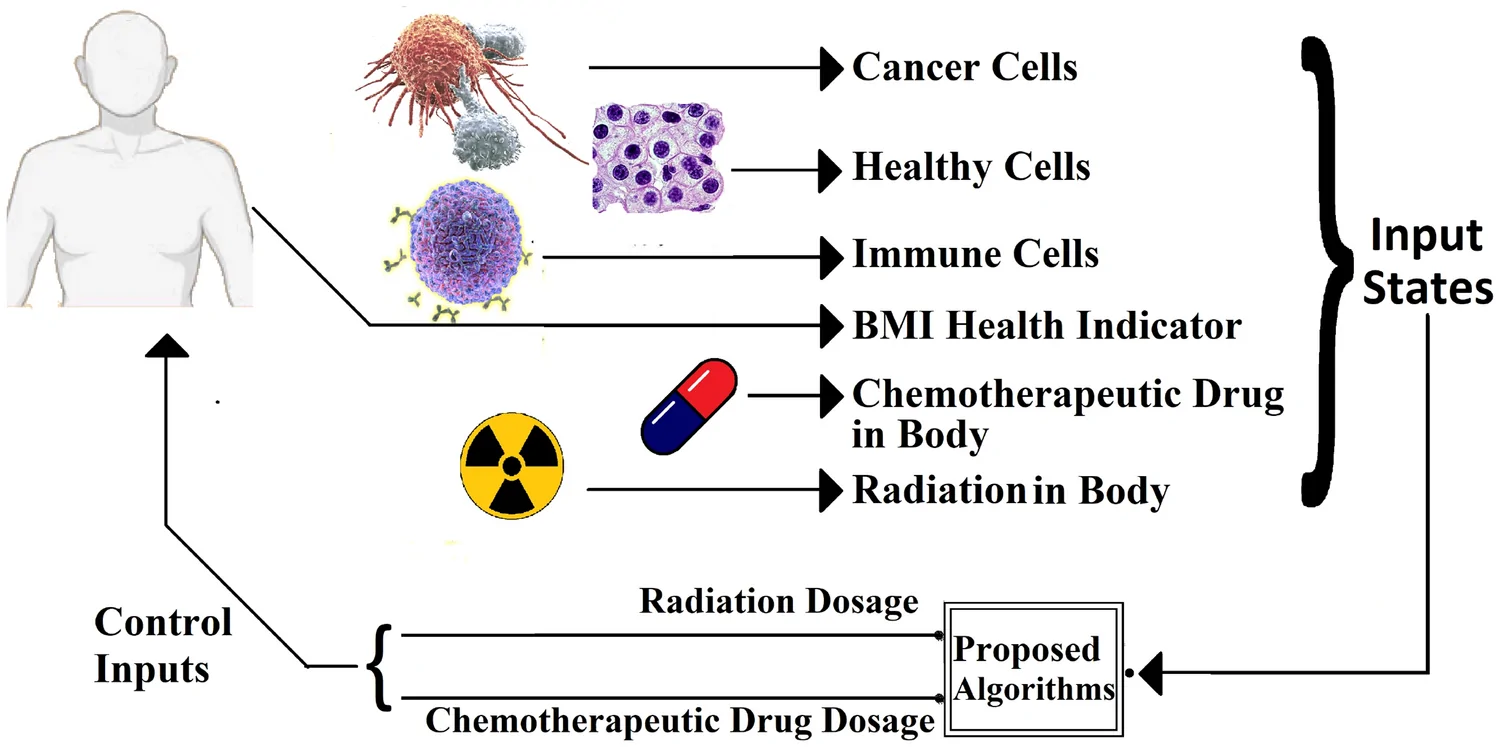
Tumor mitigation using proposed closed loop methodology.

The mathematical model presented in “Dynamical nonlinear model” has been utilized to formulate novel nonlinear algorithms and controllers that adjust the control inputs to eliminate cancerous tumors. As stated earlier in “Introduction”, there are numerous methods to directly or indirectly target and annihilate cancer cells. This study proposes a closed loop system that utilizes two most common, but effective procedures to hamper tumor growth, i.e. radio and chemotherapy. The closed loop process for radiation and chemotherapeutic drug dosage control has been described in Fig. 1. The motivation behind this research study is to present a methodology that not just combines radio and chemotherapy but also governs the feedback process to automatically adjust the control input dosages, while ensuring safety, with maximum reduction of tumor proliferation. The mathematical model that describes tumor growth, interaction of tumors cells with the human immune system, as well as radiation and chemo dosages is highly nonlinear. Thus, the first choice for managing tumor development is the use of robust nonlinear SMC. However, SMC algorithm inherently produces chattering in control inputs, resulting in undesirable system state dynamics. Thus utilizing SMC for this sensitive application of tumor reduction may result in adverse consequences and pose a health risk for the patient. Thus, to mitigate this issue, sigmoid function based control has been proposed to be incorporated into the infrastructure of SMC. As a smoothing function, this sigmoid-based control will remove SMC related inherent transients without compromising the robustness of the algorithm. The design of the sigmoid-based SMC and its stability proof has been provided in “Sigmoid function-based SMC”.

Although SMC has numerous advantages, the controller itself is unable to find the best possible combination of the two considered control inputs. Even though the control inputs are smoothed out, the inherent tendency of SMC to converge the trajectories of the system to the sliding surface, as fast as possible, results in a fairly high amount of remedial dosages. As high dosages of radiation and chemotherapeutic drugs are more toxic and have adverse impacts on patient health, it is pertinent to develop techniques that provide optimized dosages, that cause minimal toxicity while minimizing tumor cell population and maximizing patient health indicators.

To incorporate such constraints to determine optimized radiation and chemotherapeutic drug dosages, a RL-based optimization algorithm upgraded with sigmoid SMC is proposed in “Sigmoid SMC-based PPO algorithm”. Just like optimal control algorithms, artificial intelligence or ML based techniques are also capable of incorporating complex constraints while optimizing drug inputs by employing a cost or objective function. Moreover, reinforcement learning algorithms can support data-driven action selection, and once trained, do not require explicit analytical information of system dynamics at each decision step. Hence, the RL-based PPO algorithm is upgraded by incorporating sigmoid function-based SMC to determine optimized dosages of the control inputs.

### Control objectives

As discussed in “Introduction”, all of the recent studies only focused on swift tumor mitigation, without considering the harmful impacts and over-dosage proposed by those studies. Although, tumor mitigation is the pivotal aim of this study as well, however, achieving it while minimizing the toxic side effects of the treatment therapies is the real and remarkable accomplishment. The main considered objectives while designing the anti-tumor algorithms are the following:

1. Tumor cell minimization: mitigation of tumor cell population (T) to zero
2. Healthy cell proliferation: stabilization and perservation of the healthy cell population
3. Smooth input regulation: smooth administration of system control inputs “*α*” (radiation) and “*q*” (chemotherapy), to ensure avoidance of any harmful transients in system states
4. Toxicity minimization: minimization of the toxic side effects of the treatment therapies while minimizing the tumor cell population robustly as well.

### Design of the proposed algorithm

The main aim of the proposed cancer therapy is to reduce or minimize the number of tumorous and cancerous cells while maximizing the number of normal healthy cells in the body. This goal is achieved by utilizing both chemotherapeutic drug and radiation dosage as control inputs. “Sigmoid function-based SMC” provides the detailed mathematical derivation, and Lyapunov theory-based stability analysis of the smooth SMC algorithm, which is then incorporated within the framework of RL-based updated PPO algorithm in “Design of the proposed algorithm”.

#### Sigmoid function-based SMC

**Fig. 2:**
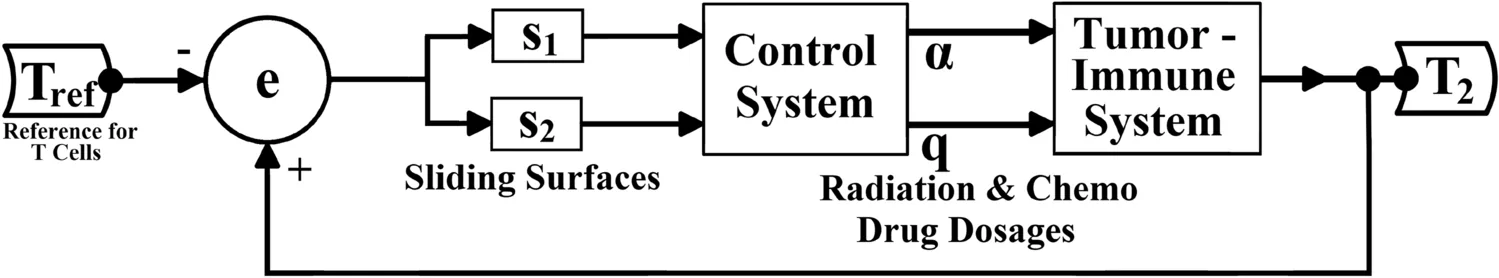
Closed loop control using multi-input smooth SMC.

SMC has numerous advantages over other nonlinear controllers. It is robust and can cater to non-linearities and perturbations more effectively. Moreover, it is simpler and easier to design and implement. However, owing to the inherent behavior of SMC operation, chattering is introduced in system’s response which may sometimes become problematic^31^. For the particular application of drug regulation or radiation control, SMC’s robust response with chattering can become a nuisance. As rapid transitions in the values of treatment variables can create medical issues, a sigmoid based smoothing function is proposed to be introduced in SMC control to smooth out its inherent rapid transients. The control loop utilized by the proposed SMC is depicted in Fig. 2.

Let *e* be the error term defined as the error between actual and desired number of tumor cells. As the latter is 0, thus:

2 $$\begin{aligned} e = T - 0 \end{aligned}$$

Since the system has multiple control inputs, two different sliding surfaces are considered here to compute the control inputs *α* and *q*. Consider the following sliding surfaces and their derivatives:

3 $$\begin{aligned} s_1&= c_1e + c_2U \end{aligned}$$

4 $$\begin{aligned} s_2&= c_1e + c_3P \end{aligned}$$

5 $$\begin{aligned} \dot{s}_1&= c_1\dot{e} + c_2\dot{U} = c_1\dot{T} + c_2\dot{U} \end{aligned}$$

6 $$\begin{aligned} \dot{s}_2&= c_1\dot{e} + c_3\dot{P} = c_1\dot{T} + c_3\dot{P} \end{aligned}$$

where, $c_1$, $c_2$ and $c_3$ are positive constants. To bring the system trajectory to the sliding surfaces and then to keep it there all the time, let:

7 $$\begin{aligned} \dot{s_1}&= c_1\dot{T} + c_2\dot{U} = \frac{-ks_1}{1+e^{-\phi s_1}} \end{aligned}$$

8 $$\begin{aligned} \dot{s_2}&= c_1\dot{T} + c_3\dot{P} = \frac{-ks_2}{1+e^{-\phi s_2}} \end{aligned}$$

where, *k* is a positive definite design constant. The parameter *ϕ* reflects steepness of the sigmoid function. The greater the value of *ϕ* is, the steeper is the sigmoid function.

Now, to analyze the stability of the system, consider the following Lyapunov candidate functions:

9 $$\begin{aligned} V_1&= \frac{1}{2} {s_1}^2 \end{aligned}$$

10 $$\begin{aligned} V_2&= \frac{1}{2} {s_2}^2 \end{aligned}$$

By taking the time derivative of Eqs. (9) and  (10):

11 $$\begin{aligned} \dot{V_1}&= s_1\dot{s}_1 = -s_1(\frac{ks_1}{1+e^{-\phi s_1}}) \end{aligned}$$

12 $$\begin{aligned} \dot{V_2}&= s_2\dot{s}_2 = -s_2(\frac{ks_2}{1+e^{-\phi s_2}}) \end{aligned}$$

Hence, Eqs. (11) and  (12) can be written as:

13 $$\begin{aligned} \dot{V_1}&= s_1\dot{s}_1 = -(\frac{k(s_1)^2}{1+e^{-\phi s_1}}) \end{aligned}$$

14 $$\begin{aligned} \dot{V_2}&= s_2\dot{s}_2 = -(\frac{k(s_2)^2}{1+e^{-\phi s_2}}) \end{aligned}$$

Since, *k* is a positive constant, and $1/(1+e^{-x})$ is always *≥* 0 for all values of *x*, $\dot{V}_{1}$ and $\dot{V}_{2}$ are proven to be negative definite, which ensures that the error stably converges to zero. As the Lyapunov candidate functions ${V}_{1}$ and ${V}_{2}$, considered to analyze the stability of the closed-loop system are positive definite. Whereas, their time derivatives $\dot{V}_{1}$ and $\dot{V}_{2}$ are proved to be negative definite in Eqs. (13) and  (14). Thus, ensuring boundedness of the system states and asymptotic convergence towards the sliding surface.

Updated SMC’s control laws can now be computed via Eqs. (7) and (8). The two control inputs *α* and *q* can be computed as:

15 $$\begin{aligned} \alpha&= \frac{c_1}{c_2}(UT - r_1T(1 - \frac{T}{k_1}) + a_{12}NT + a_{13}TI) + \gamma U - \frac{k}{c_2}(\frac{s_1}{1+e^{-\phi s_1}}) \end{aligned}$$

16 $$\begin{aligned} q&= \frac{c_1}{c_3}(N_T(1-e^{-P})T - r_1T(1 - \frac{T}{k_1}) + a_{12}NT + a_{13}TI) + \sigma P - \frac{k}{c_3}(\frac{s_2}{1+e^{-\phi s_2}}) \end{aligned}$$

Here, the terms $- \frac{k}{c_2}(\frac{s_1}{1+e^{-\phi s_1}})$ and $- \frac{k}{c_3}(\frac{s_2}{1+e^{-\phi s_2}})$ in Eqs. (15) and  (16) are the sigmoid based switching control inputs for *α* and *q*, whereas the remaining terms are collectively called the equivalent control. Owing to these soft switching input terms, the proposed SMC controller produces almost negligible chattering in the output response.

**Fig. 3:**
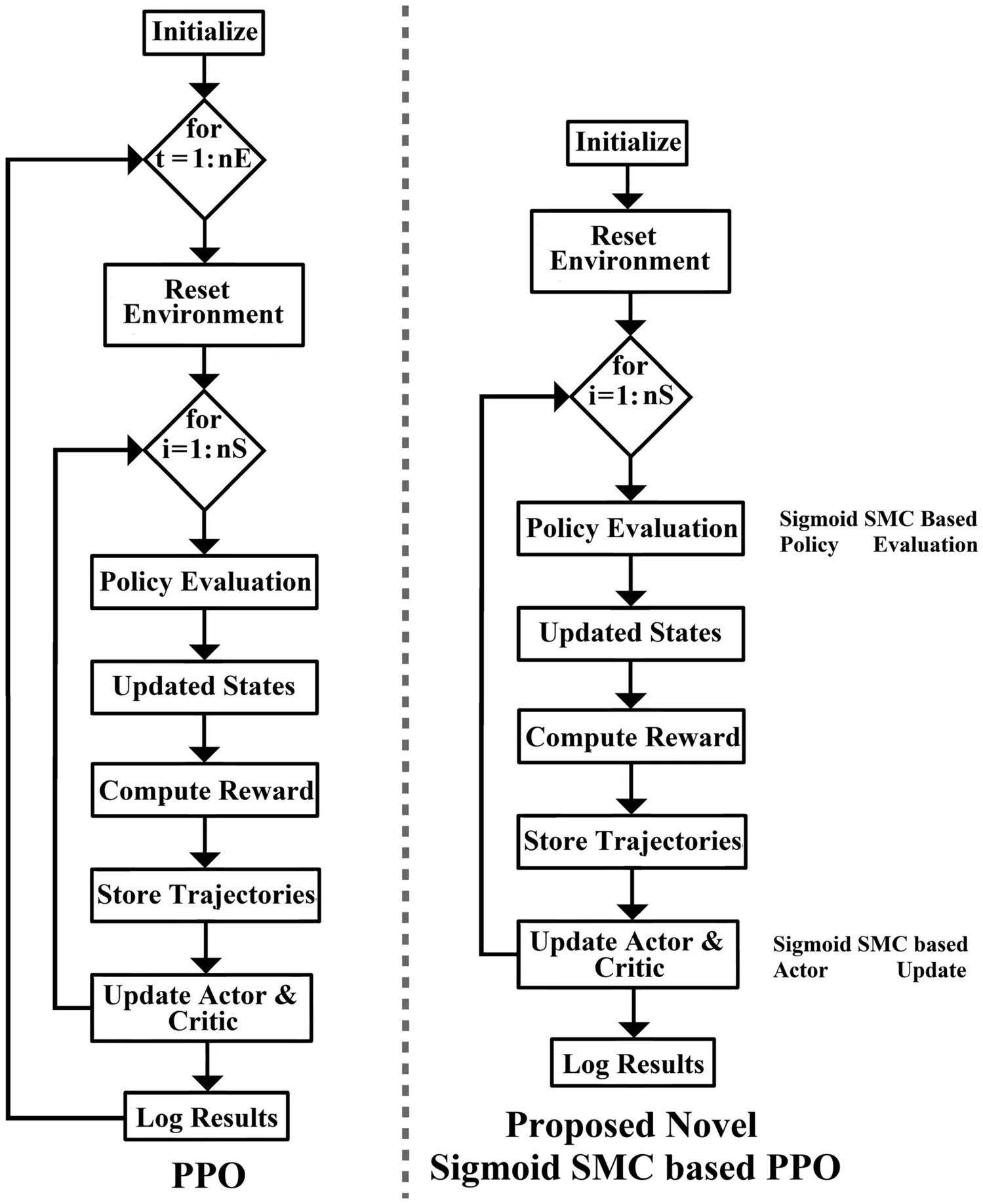
PPO and proposed hybrid smooth SMC-PPO algorithm.

#### Sigmoid SMC-based PPO algorithm

PPO is a policy gradient method belonging to the field of reinforcement learning. The algorithm optimizes a policy that ensures the minimization of a reward based objective function by taking actions and then analyzing the impact of these actions on the overall system states. The algorithm optimizes the policy, ensuring that the policy parameters are updated while remaining within a safe limit^47–49^. By combining smooth SMC with PPO, both the robustness of SMC and the learning capabilities of PPO can be exploited. The reward for each action is determined via the following equation:

17 $$\begin{aligned} reward = -w_T T^2 + w_N\times N^2 - w_I\times I^2 - w_{\alpha q}\times ({\alpha }^2 + q^2) \end{aligned}$$

where, $w_T$, $w_N$, $w_I$, $w_{\alpha q}$ are all positive constants or weights, whose values have clinical relevance. For instance, a high value for $w_T$ emphasizes the speedy reduction in tumorous cell population as it is the most critical control objective. Similarly, the negative sign with the terms such as $T^2$ implies that the reward will decrease with an increase in that particular quantity (which is tumor cell population over here). Based on the cost or objective function provided in Eq. (17), the reward will increase if the tumor population is reduced or if the healthy cell population is increased. Similarly, an increase in the computed radiation and chemotherapeutic drug inputs implies a corresponding decrease in the reward. Thus, for the algorithm to increase the reward after each successive computation of the control inputs, the algorithm has to minimize the tumor cell population, and it has to maximize the healthy normal cell proliferation. Similarly, the algorithm has to minimize administered dosages of radiation and chemotherapeutic drug to limit the toxicity caused by the proposed treatment procedure. The original and upgraded PPO process as well as proposed novel sigmoid SMC based PPO processes are explained in more detail in Fig. (3).

Pseudo-code: The proposed hybrid smooth SMC-PPO algorithm can be summarized in the following steps:

- Initialize the actor and critic weights
- Set the states to their initial values according to the health conditions of the patient
- Reset system to initial state
- For each time-step, do:
  1. Generate control actions using current actor policy
  2. Apply action to nonlinear tumor dynamics
  3. Observe next state and immediate reward
  4. Store state-action-reward trajectory
- Estimate returns and advantages from collected trajectory
- For each time-step, do:
  1. Compute smooth sliding surface based on tumor state
  2. Compute smooth SMC control signal
  3. Update actor parameters using PPO Objective (advantages shaped by smooth SMC signal)
  4. Update critic parameters using value error minimization
- Return learned hybrid control policy

This study integrates the sigmoid based SMC with PPO by altering two blocks of the original algorithm as mentioned in Fig. 3. The proposed algorithm integrates smooth SMC into PPO learning framework by shaping the policy update with a robust nonlinear control signal derived from tumor dynamics. First, the policy evaluation block, which provides the updated actions (or control inputs) to be applied to the system, incorporates sigmoid-based SMC control laws derived in “Sigmoid function-based SMC”. However, this policy (or control inputs) is not just formulated on the basis of smooth SMC only. It is also dependent on the “actor weights” (for more information on PPO, readers are recommended to read:^47–49^), which are determined in the update actor and critic blocks. The second instance where the smooth SMC terms are incorporated within the PPO framework is to the “update actor and critic” block itself. This block or function in original PPO algorithm utilizes probabilistic approach to calculate actor and critic weights that slowly and steadily allows the system trajectories to move towards the desired state. This study modifies the process of actor weights calculation by incorporating the smooth SMC terms so that the robustness of SMC may help the algorithm to reach the desired state efficiently and robustly.

This study exploits the inherent characteristic of PPO to “clip” overly large upgrades in actor and critic weights to ensure that the policy updates are not too aggressive, which in turn ensures smoothness of the radio and chemo dosages evaluated by the algorithm. Moreover, by including one simple change in the original PPO algorithm, the computational complexity of the technique can be reduced to *O*(*n*) from $O(n^2)$. The original algorithm updates the actor and critic weights after the conclusion of each episode, however, the upgraded algorithm incorporates this update into weights within the inner loop that runs for the number of designated time steps as depicted in Fig. 3. By doing so, the algorithm optimizes actor and critic weights, thus, minimizing tumor cells efficiently within a single episode while reducing the complexity of proposed algorithm as well.

### Sensitivity and robustness considerations

This study analytically assesses the sensitivity of system dynamics to model parameters through inspection of the governing equations and the structure of control law. The tumor growth rate ($r_1$) and carrying capacity ($k_1$) directly influence tumor dynamics through logistic growth, while immune related parameters (such as $r_3$ and $k_3$) modulate the magnitude and saturation of immune activation. Similarly, chemotherapy and radiation related parameters affect system behavior monotonically through drug and radiation induced cyto-toxicity and toxicity terms. Importantly, the proposed control strategy utilizes sigmoid function-based smooth sliding mode control (SMC), which is well known for its robustness to bounded parametric uncertainties and external disturbances. The sliding surface design ensures asymptotic convergence of system trajectories towards desired manifolds (as proven in “Sigmoid function-based SMC”) despite moderate variations in biological parameters. When combined with adaptive policy optimization of PPO, the resulting SSMC-PPO framework mitigates sensitivity to parameter perturbations without requiring precise parameter tuning. These analytical considerations support the expected robustness of the proposed framework under bounded model perturbations. Nevertheless, extensive numerical uncertainty quantification and patient-specific sensitivity analysis remain necessary future steps before translational or clinical interpretation.

### Ethics statement

This study is purely computational and simulation-based, and no human or animal data is utilized. Therefore, this study is exempt from ethical approval requirements.

## Simulation setup

This study considers the following initial conditions of the system states throughout the simulations: $[T_0; N_0; I_0; U_0; P_0; H_0] = [0.9;0.5; 0.5;0;0;25]$. As it can be observed, the healthy cell population is deliberately kept low, while considering a fairly high tumor cell population, so that the proposed control algorithms may be tested and validated more effectively.

### Environment

All simulations in this research study are performed in MATLAB using a continuous-time numerical integration framework, providing a reproducible computational modeling environment for controller evaluation and comparison. The non-linear tumor dynamics presented in Eq. (1) are solved using the built-in ode45 solver, which is based on explicit Runge-Kutta (4, 5) method and is well suited for non-stiff ordinary differential equations. For the simulation, default solver tolerances were utilized, with a relative tolerance of $10^{-3}$ and an absolute tolerance of $10^{-6}$. The modified SSMC-PPO algorithm is implemented using lightweight linear actor-critic architecture to ensure numerical stability and computational efficiency. The input layer consists of six state variables representing tumor, immune, and healthy cell populations and their associated system states. The actor network outputs two control actions, corresponding to radiation and chemotherapy dosages, while the critic estimates the state-value function. The discount factor was set to 0.99 to balance long-term tumor suppression with short-term treatment effects. PPO policy updates are stabilized using a clipping ratio of 0.1. The learning rate is selected to be 0.0001, with gradient clipping applied to prevent excessive parameter updates. The episode length consisted 101 steps, whereas, the convergence of the learning process is determined based on stabilization of the cumulative reward and consistent policy behavior.

**Table 2:** Parameter values.

Parameter	Value	Parameter	Value
$c_1$	0.1	$w_T$	1
$c_2$	1	$w_N$	0.1
$c_3$	1	$w_I$	0.05
k	5	$w_{\alpha q}$	0.1
*ϕ*	0.5		

### Controller design parameters

The design parameters of the controllers and algorithms proposed in this study along with the weights of objective function mentioned in Eq. (17) are given in Table 2. These design parameters are set to not only minimize the tumor cell population swiftly, but also to minimize the toxicity and transients produced by the control inputs.

### Performance comparison framework

All the proposed controllers are evaluated extensively based on the following metrics:

1. Baseline dosage reduction: reduction in initially administered radiation and chemo-drug dosages^30^.
2. Faster tumor convergence: time taken by the algorithms to converge tumor population to zero^2,30,50^.
3. Treatment intensity reduction: reduction in the overall chemotherapeutic and radiation drug dosages administered throughout the whole therapy^2,30,50^.

Apart from these quantitative comparison parameters, the performance of the proposed controller is also compared qualitatively with that of the recently proposed algorithms. These parameters include:

1. Smooth application of control inputs and consequently smooth system dynamics^29^
2. Computational complexity of the considered algorithm or controller^7,10^.
3. Metronomic flexibility or administration of flexible treatment dosages for tumor mitigation^39^.
4. Overall negative impact of treatment therapies on health^2,50^.
5. Input convergence to zero after successfully decimating the tumor cell population^30^.
6. Type of therapies employed for tumor mitigation^10,30^.
7. Settling time^2,50^

### Benchmarking

The comparative analysis based on the metrics mentioned above in subsection "Performance comparison framework" is performed to establish the effectiveness and superiority of the proposed algorithm. Performance of the proposed hybrid SSMC-PPO algorithm is compared with that of the following recently proposed control techniques: multi-input optimal control, SMC, Synergetic, state-feedback, fuzzy, PID, supertwisting algorithm and deep reinforcement learning twin delayed deep deterministic policy gradient algorithm.

**Fig. 4:**
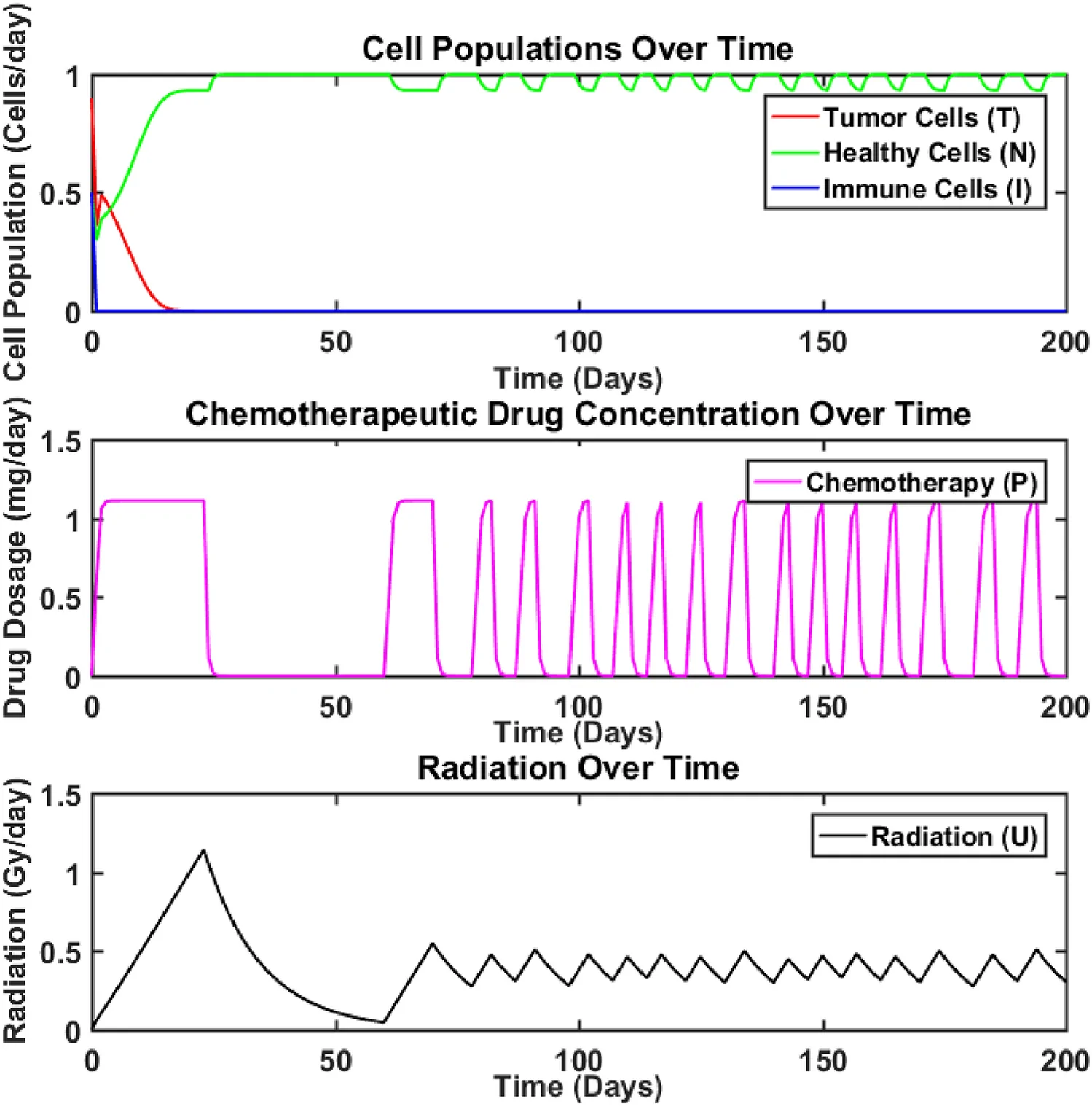
System dynamics using SMC.

## Results

This section analyses and compares the performance of proposed novel algorithm. To establish its superiority, the performance of the proposed hybrid SSMC-PPO has been compared in this section with conventional SMC, sigmoid function-based smooth SMC, and PPO algorithm. The proposed model (Eq. 1) has been utilized to study the response and dynamics of all these controllers for the application of tumor mitigation. Firstly, “Conventional SMC vs sigmoid function-based smooth SMC” presents the system and control input dynamics when conventional SMC and sigmoid function-based smooth SMC are utilized. Whereas, “ PPO vs sigmoid SMC-based hybrid PPO” highlights the system and control input dynamics under the impact of original PPO algorithm as well as the proposed hybrid smooth SMC-based PPO (SSMC-PPO). Lastly, the “ Metronomic chemo and radiation therapy” explains a key feature of the proposed SSMC-PPO algorithm that outlines its versatility and distinctiveness in comparison with other recently proposed techniques.

### Conventional SMC vs sigmoid function-based smooth SMC

SMC is a highly robust and effective nonlinear controller, well-suited for rapidly and reliably driving the system trajectory towards the desired states. The dynamics of the system states, when SMC was utilized are depicted in Fig. 4. By employing the SMC on tumor-immune system model provided in Eq. 1, the controller swiftly mitigated the tumor cell population. SMC achieved this by administering large dosages of radiation and chemotherapeutic drug. However, as discussed in “Introduction”, this administration of high input dosages has dire consequences for the patient. Thus, even though the controller mitigated tumor population swiftly, yet it will lead to treatment related severe toxicity. Moreover, apart from swift mitigation of tumor cell population and administration of high treatment dosages, the dynamics of control inputs and system states depicted huge transients. These oscillations can also have severe clinical and psychological consequences for the patient under consideration. It is normally desired to administer these treatment dosages at levels that the body can easily adapt to. Lastly, even though the algorithm successfully mitigated the tumor cells, yet both radiation and chemotherapeutic dosages do not converged to zero by the controller.

**Fig. 5:**
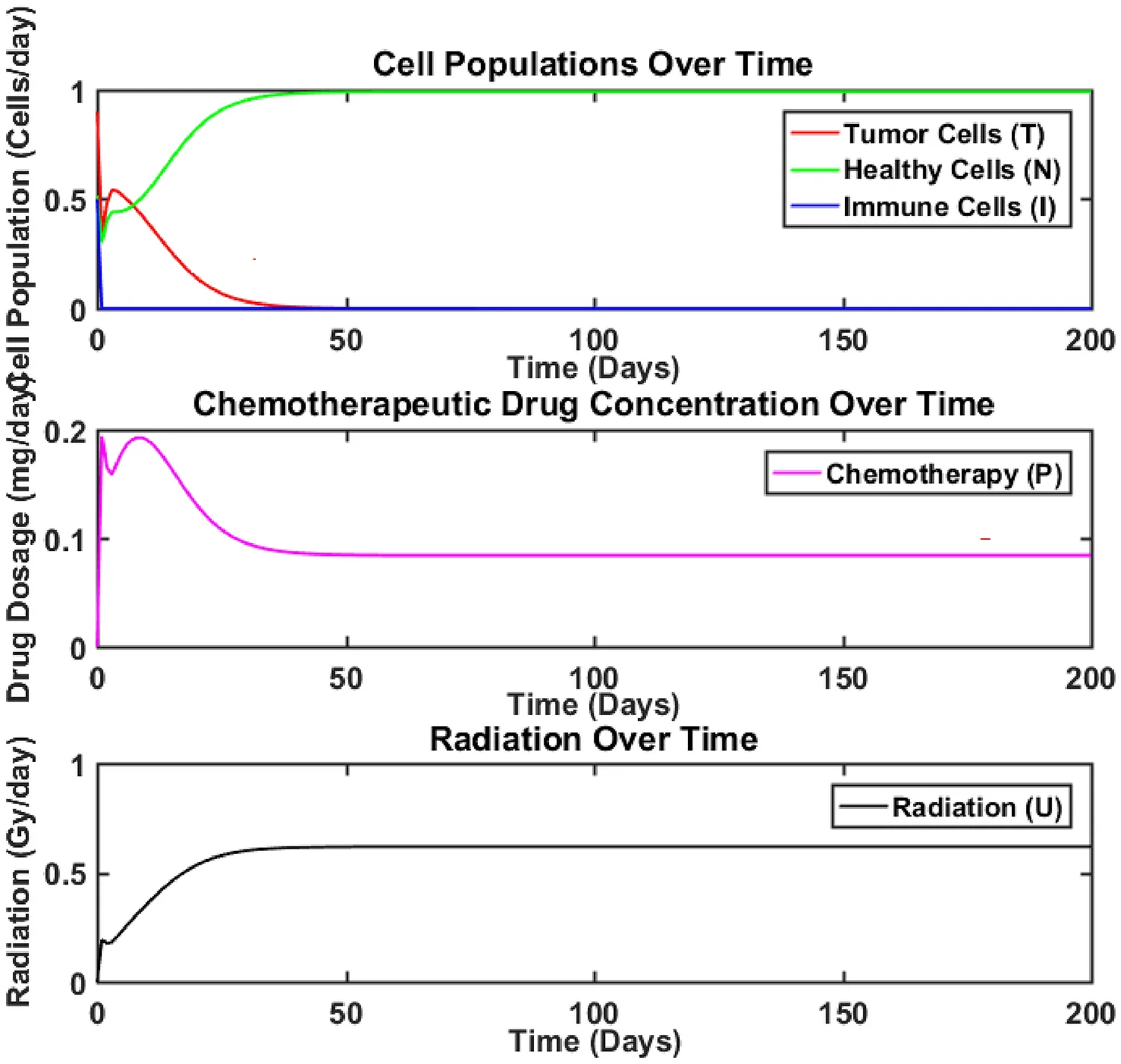
System dynamics using sigmoid function-based SMC.

The state curves obtained by applying sigmoid-based SMC, shown in Fig. 5, are more appropriate as they depict much smoother control inputs. The state dynamics obtained as a result of administering smooth treatment dosages can also be observed to be transient free. However, the disadvantage of utilizing sigmoid function-based SMC is the slow convergence rate of the system dynamics. Also, the control inputs did not converge to zero once the tumor cell population is successfully decimated. Another important observation from the control input dynamics depicted in Fig. 5 is the relatively low amount of administered dosages of radiation and chemotherapeutic drug, in comparison to that depicted in Fig. 4 by employing SMC.

**Fig. 6:**
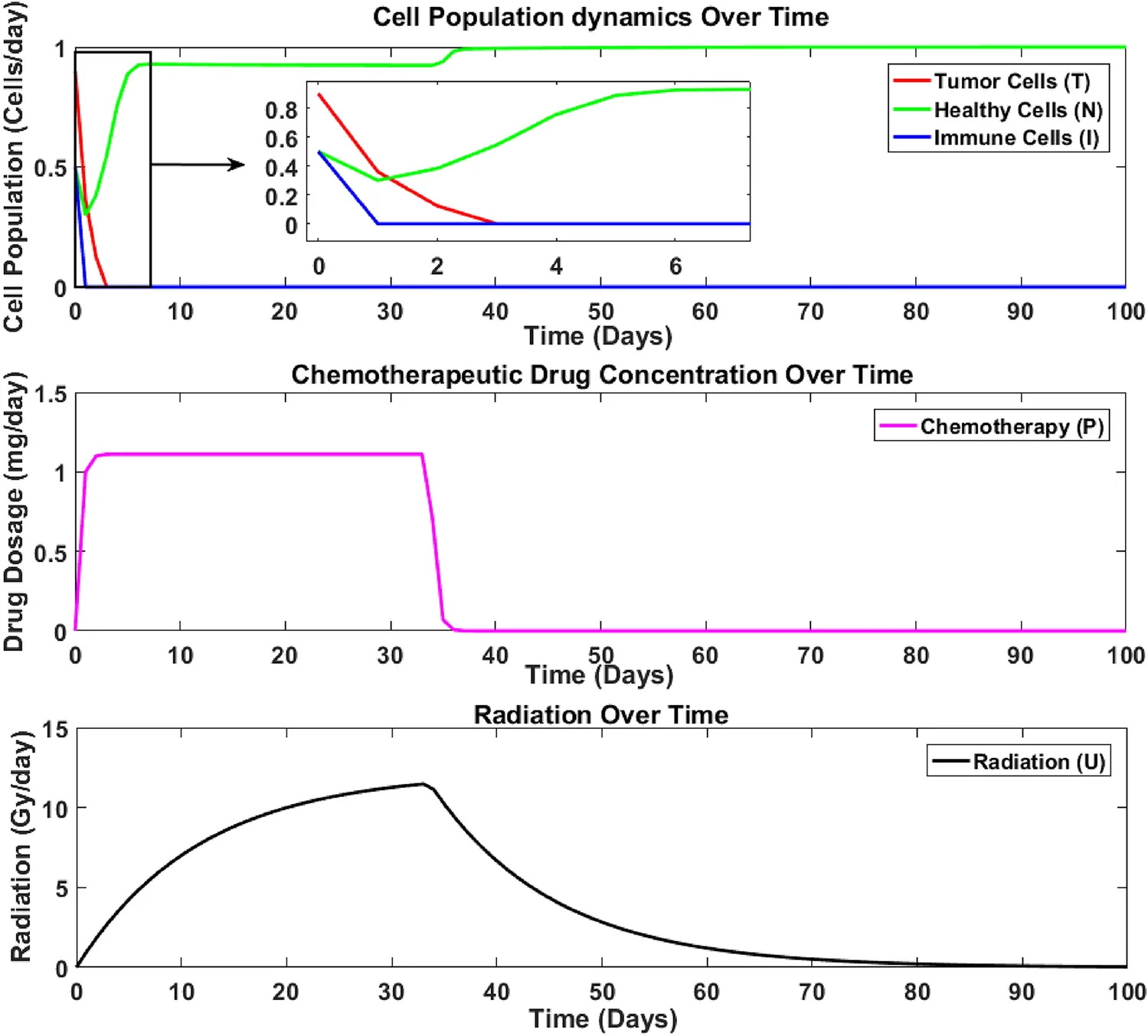
System dynamics using proximal policy optimization.

###  PPO vs sigmoid SMC-based hybrid PPO

Figure 6 depicts the system response when original PPO was employed for tumor mitigation. It can be observed that by utilizing PPO, the obtained system dynamics are smooth and transient free. The algorithm efficiently mitigated the tumor cell population while maximizing the healthy and normal cells. However, the administered dosages of radiation and chemotherapeutic drug inputs are much higher in comparison to those depicted in Fig. 5 (sigmoid-SMC). Thus, the PPO failed to manage and contain toxicity caused by the treatment procedures effectively. Similarly, even though the algorithm converged control inputs to zero, yet it kept on administering the treatment dosages even after the eradication of tumor cell population. This sluggish response of effectively managing the treatment dosages is reflected in slow recovery of healthy cells as high values of administered radiation and chemotherapeutic drug dosages have a negative impact on their proliferation.

**Fig. 7:**
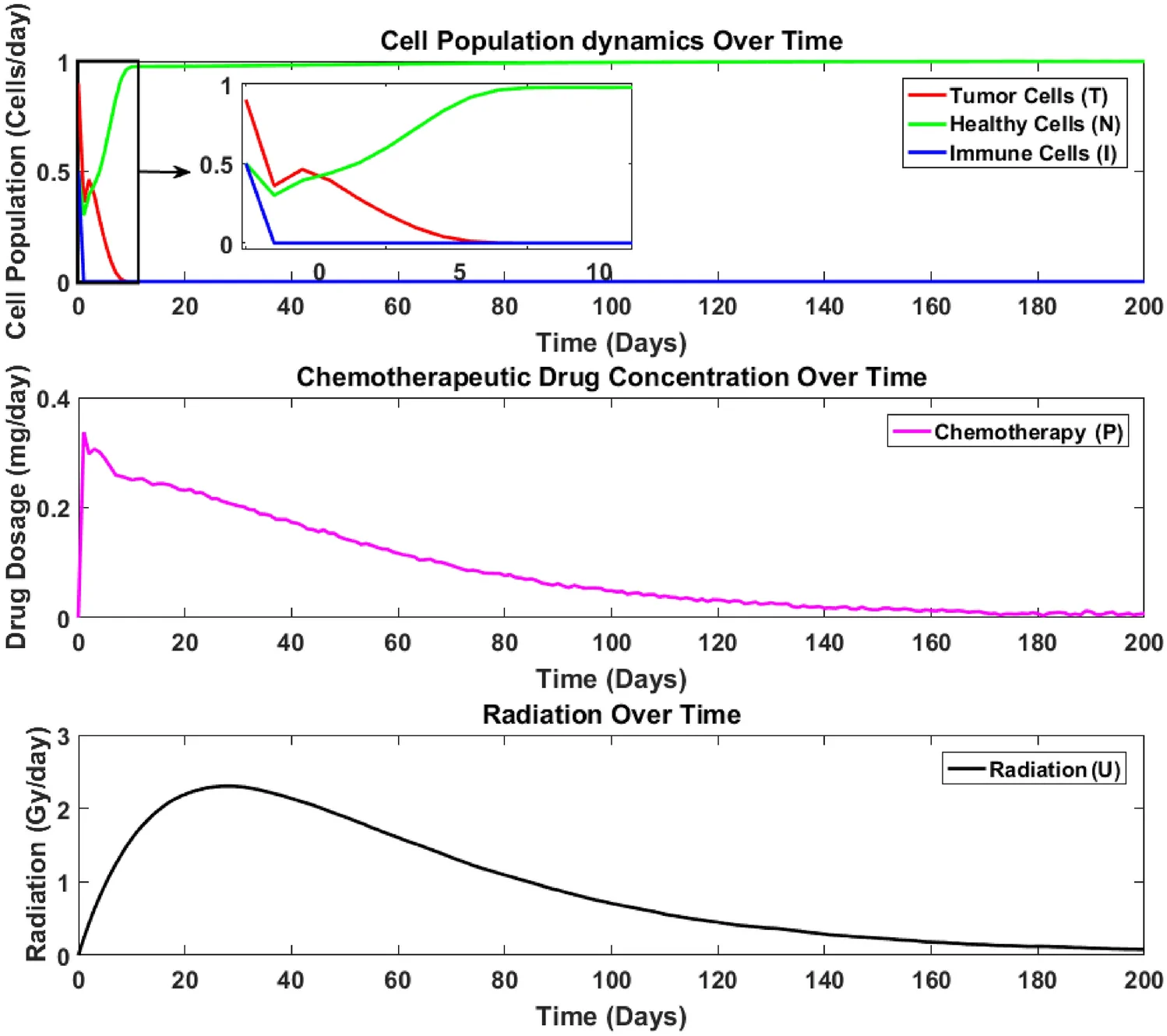
System dynamics using SSMC-PPO algorithm.

Similarly, the response of system states under proposed SSMC-PPO algorithm is depicted in Fig. 7. and 8. The system states portrayed smoothness and steadiness without any noteiceable transients. The radiation and chemotherapeutic drug dosages administered by the hybrid SSMC-PPO algorithm not only remained very low but they also converged to zero. This implies that the proposed hybrid SSMC-PPO algorithm caused the least treatment-related toxicity while successfully decimating the tumor cell population. Moreover, the system states swiftly and robustly converged to their final values inferring that the algorithm depicted smoothness of sigmoid function, robustness of SMC algorithm and, achieved multi-objective optimization successfully. Thus, the proposed algorithm is highly desirable when considering patient comfort and well-being while prioritizing safety and minimizing treatment related risks.

**Fig. 8:**
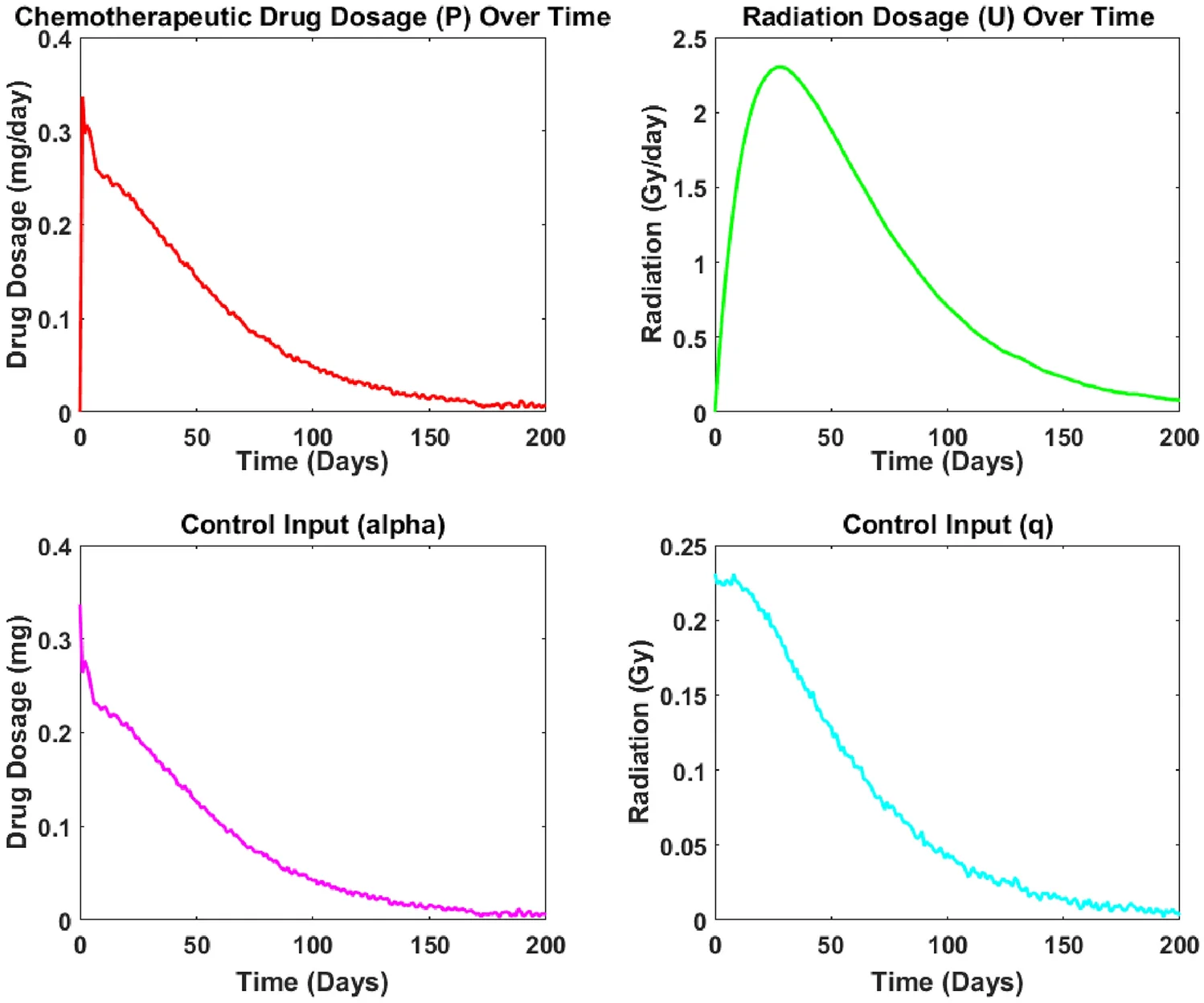
Treatment dynamics and control inputs of SSMC-PPO algorithm.

**Fig. 9:**
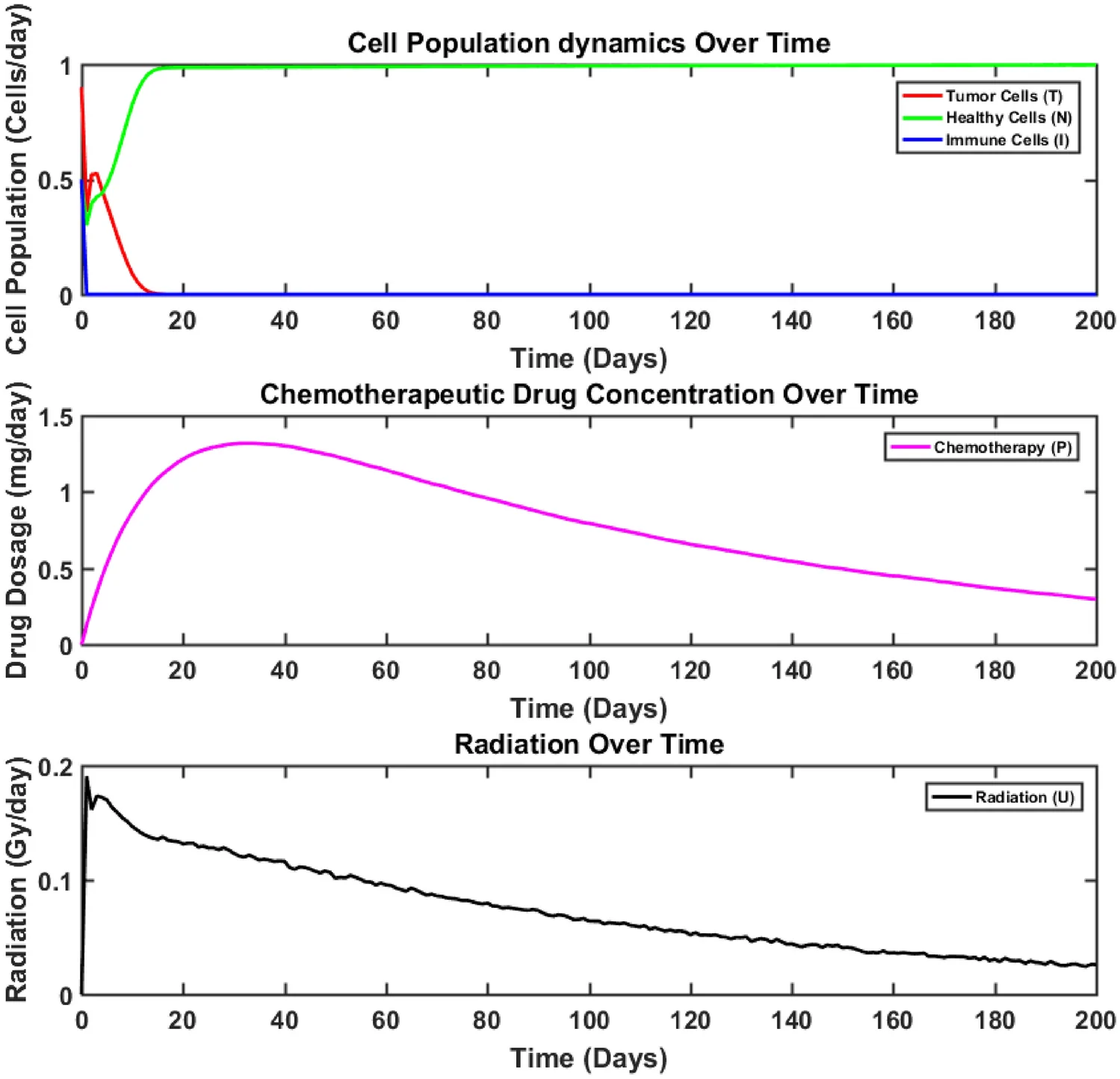
Metronomic mode: system dynamics using SSMC-PPO algorithm.

Another salient feature of the proposed SSMC-PPO algorithm is its ability to achieve control objectives without depending on the system model. Both the original PPO and proposed hybrid SSMC-PPO algorithm utilized only the mathematical model given in Eq. (1) for training and performance verification purposes. However, the “action” (or control input) determining steps of these algorithms are not dependent on the mathematical dynamics of the system. Thus, if availability of data is ensured from any other source, both PPO-based algorithms can comfortably determine the actions (control inputs) on the basis of that. As discussed in “Sigmoid function-based SMC”, the SMC control law has two parts, the equivalent control and the switching control. This study has also tested the sigmoid SMC based PPO algorithm without incorporating the equivalent control part. As the PPO utilizes its own mechanism for control input calculations, it can replace the equivalent control part of the sigmoid-based SMC to exploit benefits of both controllers. By doing so, the proposed SSMC-PPO algorithm reduces its dependence on the explicit equivalent control formulation and requires tumor-state information to compute the sliding surface and treatment actions. In this study, this property is demonstrated within the ODE simulation environment; future work with patient-derived data is required before claiming clinically model-independent deployment. The results presented in Fig. 9 have been obtained by implementing the proposed PPO-based novel algorithm without considering the equivalent control part of the sigmoid-based SMC. Moreover, the results obtained from Fig. 9 have also been simulated to prove the metronomic drug dosage capability of the proposed controller, as proposed in^39^. The metronomic flexibility of the proposed SSMC-PPO algorithm has been discussed in detail in “ Metronomic chemo and radiation therapy”.

###  Metronomic chemo and radiation therapy

**Fig. 10:**
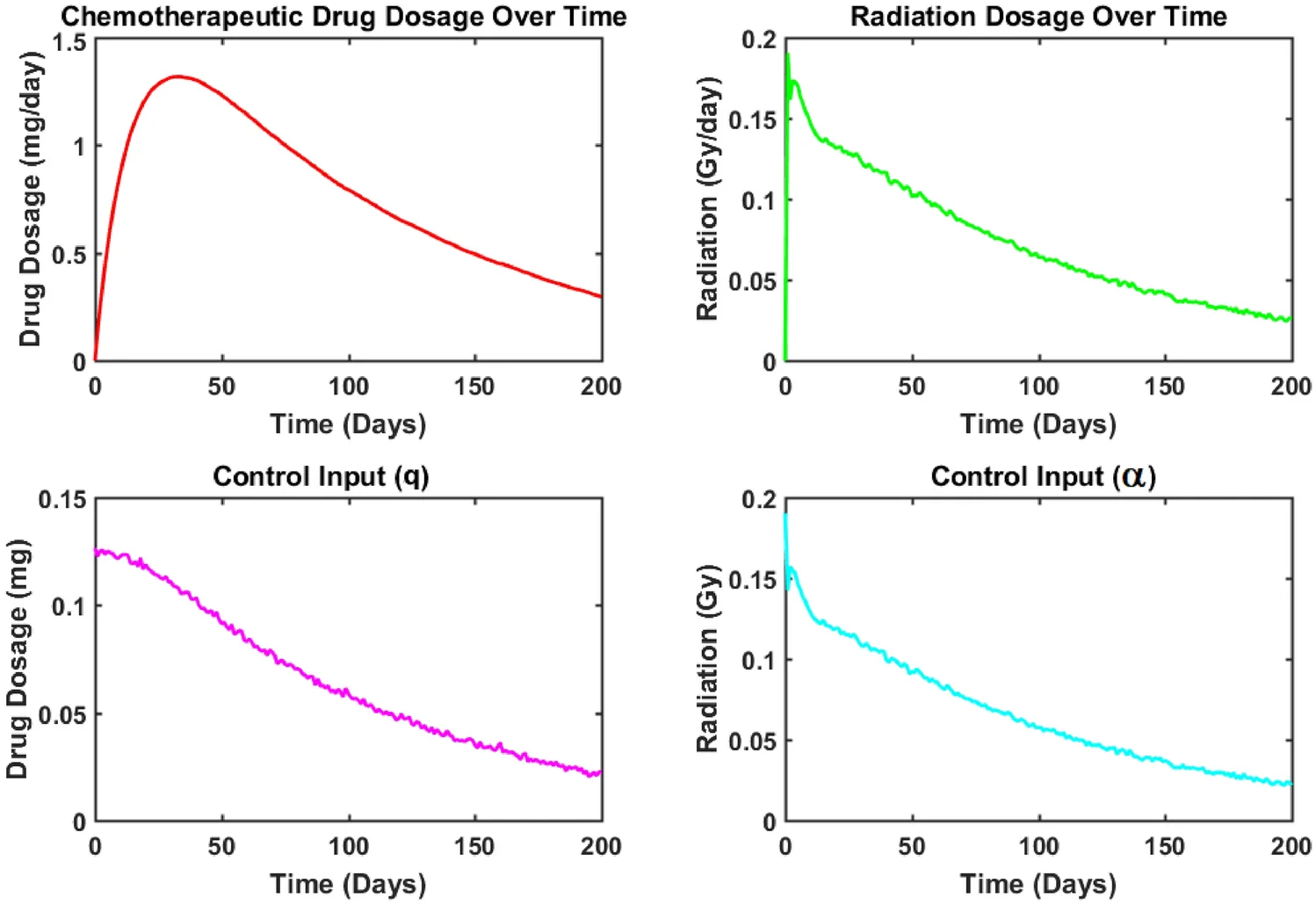
Metronomic mode: control inputs of SSMC-PPO algorithm.

Figures 9 and  10 suggest another important feature of the proposed algorithm, making it suitable for metronomic chemo and radiation therapy, in which low but frequent dosages of the treatment agents are administered to minimize the stress of anti-tumor therapies on the patient. To accommodate patient specific personalized treatment plans, the anti-tumor algorithm should be capable of adjusting the control laws to accommodate low radiation and chemotherapeutic drug inputs while continuously minimizing the tumor cell population while steadily promoting healthy cell growth. Thus, the proposed SSMC-PPO algorithm is not only robust and smooth, but is also flexible and adaptable enough to offer treatment in different modes, depending on the personalized medical situation of patients. On comparing Fig. 8 (control inputs in normal mode) with Fig. 10 (control inputs in metronomic mode), it can be observed that the magnitude of dosage as well as total dosage administered throughout the therapy is sufficiently low in the case of metronomic mode of operation. The algorithm robustly and smoothly mitigates the tumor cell population while maintaining transient free dynamics. Moreover, the control inputs converge towards zero as well.

**Table 3:** Transient analysis of controllers.

Method	State	RT (days)	FT (days)	ST 2% (days)	ST 5% (days)	SSE cells
SMC	T	–	12	16	15	0
	N	20	–	60	55	0.15
Sig-SMC	T	–	50	86	66	0
	N	58	–	99	70	0.007
PPO	T	–	3	5	3	0
	N	5.5	–	33	32	0
SSMC-PPO	T	–	5	7	6.8	0
	N	9	–	37	9	0

*RT* rise time, *FT* fall time, *ST* settling time, *SSE* steady state error, *SMC* sliding mode control, *Sig-SMC* sigmoid function-based sliding mode control, *PPO* proximal policy optimization, *SSMC-PPO* sigmoid sliding mode control-based proximal policy optimization.

### Achieved results

**Fig. 11:**
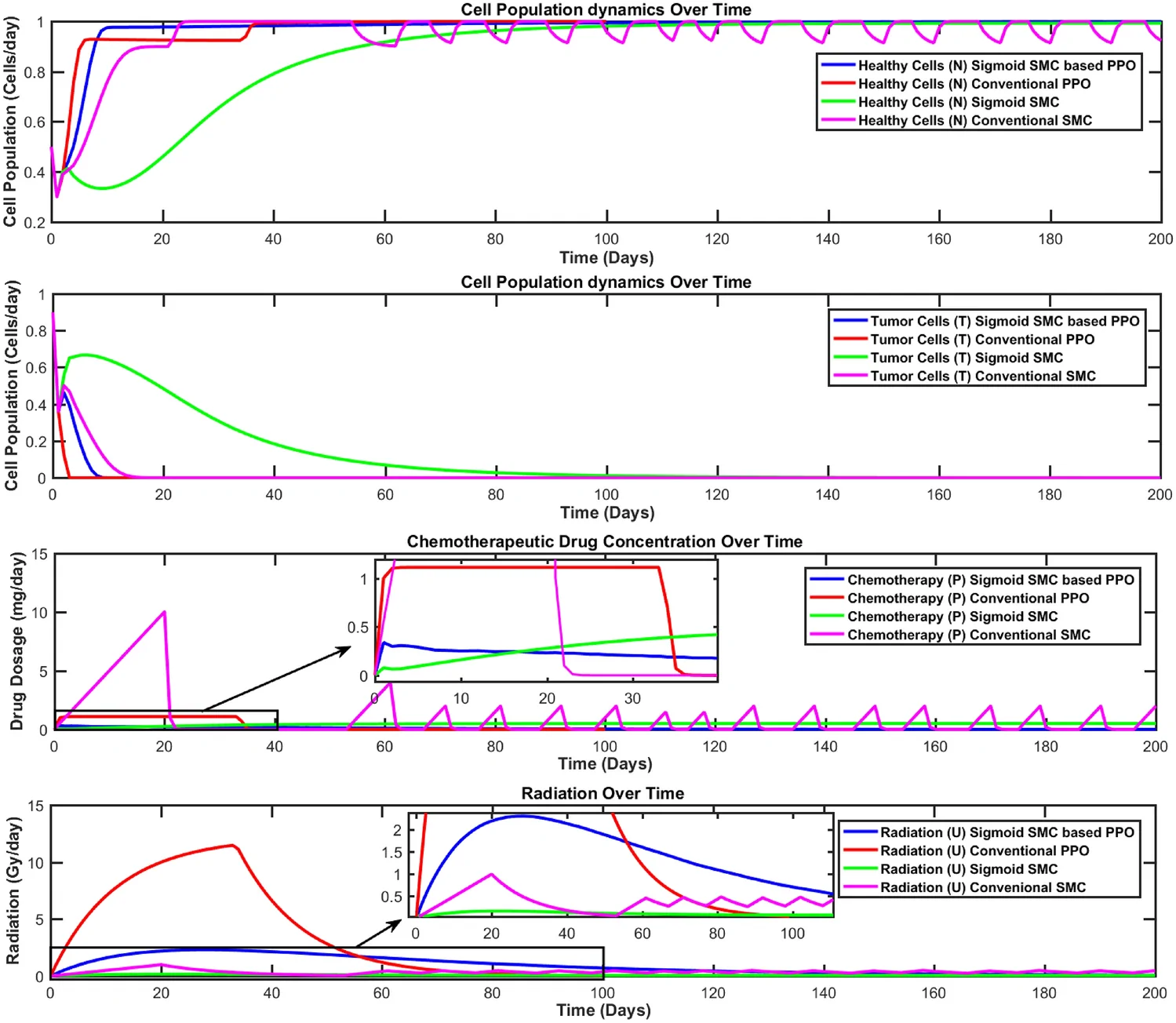
Comparison between proposed sigmoid SMC based PPO vs others.

The observations and results, discussed in “Conventional SMC vs sigmoid function-based smooth SMC” and “ PPO vs sigmoid SMC-based hybrid PPO” are comprehensively presented in tabular form in Tables 3 and 4. Similarly, the results presented in “Conventional SMC vs sigmoid function-based smooth SMC” and “ PPO vs sigmoid SMC-based hybrid PPO” are illustrated again in Fig. 11 to provide a clearer depiction of the performance of proposed hybrid SSMC-PPO algorithm. Table 3 analyzes the transient response of the proposed SSMC-PPO algorithm and compares it with the performance of conventional SMC, smooth SMC and original PPO algorithm. Apart from transient analysis, there are many important attributes and characteristics that an algorithm must possess, which are generally not focused upon in recent literature. This study has included some of these parameters, mentioned in Table 4, to present a more realistic and compelling comparison. The comparison established in Table 3, is based on rise time (RT) or fall time (FT), settling time (ST; 2% and 5 % criteria) and observed steady state error (SSE). The rise time is considered to be the minimum time taken by the dynamics to reach 90% of its final value. Similarly, the settling time is considered to be the time (in days) required by the dynamics to either reach 2% or 5% of their final steady state value. The negative impact on health by each considered algorithm is evaluated through a combined assessment of (i) the transients in system states, (ii) the ability of control inputs to converge to zero after successful tumor mitigation, (iii) the flexibility of each algorithm to administer chemotherapy and radiation in a metronomic (low dose, sustained) manner, (iv) the overall intensity of chemo and radiation dosages throughout the treatment horizon, and (v) the observed dynamics of state “H”. Algorithms that achieved tumor suppression with smoother control profiles, lower cumulative dosages, faster attenuation of control actions, and reduced disruption to the health dynamics are classified as having a lower negative impact on health. Similar criteria are also observed to evaluate this negative impact on health in “Discussion: proposed sigmoid SMC-based PPO vs. algorithms proposed in recent studies” for each considered algorithm.

**Table 4:** Controller performance comparison.

Method	Smooth response	Model dependence	Comp. complexity	Metronomic flexibility	Negative impact on health	Input conv. to 0
SMC	No	Yes	*O*(*n*)	No	Very high	No
Sig-SMC	Yes	Yes	*O*(*n*)	No	High	No
PPO	Partial	No	$O(n^2)$	Yes	High	Yes
Sig-SMC-PPO	Yes	No	*O*(*n*)	Yes	Minimal	Yes

*SMC* sliding mode control, *Sig-SMC* sigmoid function-based sliding mode control, *PPO* proximal policy optimization, *SSMC-PPO* sigmoid sliding mode control-based proximal policy optimization. To ensure fair comparison, all controllers in Table 4 were evaluated within the same ODE-based tumor-immune model and multi-modal therapy framework. For each experiment, the same model parameters, simulation conditions, and initial conditions were applied consistently across all considered controllers, and the same comparison criteria were used to assess smoothness, model dependence, computational complexity, metronomic flexibility, impact on health, and input convergence

Similarly, the performance comparison is established in Table 4 on the basis of six metrics. The first parameter is the smoothness in obtained dynamics of tumor and healthy cells, which is essentially dependent on the smoothness in control inputs. The second parameter indicates whether an algorithm is dependent on the mathematical model of the system or not, whereas, the third parameter is the computational complexity of the respective algorithm. The 4th parameter of comparison is based on the ability of controller to administer metronomic control inputs. The next parameter is based on the state *``H''* and explains the adverse impacts of radiation and chemotherapeutic drugs on the general health and well-being of patients undergoing proposed treatment. The extremely high values indicate that the health of the patient under consideration has deteriorated the most due to the excess administration of radiation and chemotherapeutic drugs. The last parameter of comparison is the ability of considered algorithm to converge the control inputs to 0 after successfully decimating the tumor population.

By analyzing Table 3, it can be observed that both sigmoid-based controllers generally proved to be a bit sluggish in comparison to their non-smooth transient prone counterparts. Although, the original PPO algorithm depicted very swift dynamics, yet it achieved this robustness by administering very large input dosages. This leads to soaring of settling time for the healthy normal cells (mentioned as “N” in Table 3) as administration of high treatment dosages have a negative impact on the growth and proliferation of healthy cells. On the other hand, the proposed SSMC-PPO algorithm mitigated tumor cell population and took almost similar rise time *\backslash* fall time and settling time, to that of original PPO algorithm. And this feat is achieved, while administering relatively much lower dosages of radiation and chemotherapeutic drug. Lastly, slight steady state errors can be observed when SMC and sigmoid function-based SMC were employed for tumor mitigation. Whereas, no such errors are observed when original PPO or proposed hybrid SSMC-PPO algorithms were utilized.

The transient analysis related parameters can reflect the robustness and efficiency of an algorithm, yet, for a more detailed performance analysis, a comprehensive qualitative comparison is provided in Table 4. Even though SMC is one of the most robust nonlinear controllers, yet it depicted relatively large transients. Moreover, after the successful mitigation of tumor cell population, the control inputs did not converge to zero, indicating that the controller causes toxicity for the patient and is thus not appropriate for this sensitive application of tumor reduction through automatic control. Similarly, the SMC algorithm allows administration of high dosages of radiation and chemotherapeutic drug to curb the tumor advancements instantly, resulting in increased toxicity that may become unbearable for the ailing patient under consideration. A similar conclusion can be made by assessing the performance metrics for sigmoid function-based smooth SMC algorithm. The sigmoid SMC algorithm depicted transient free dynamics and resulted in a bit less toxic treatment procedure than conventional SMC. But both the controllers were unable to converge their administered control inputs to zero. Moreover, both the controllers are not much flexible to adjust the treatment dosages according to the personalized medical conditions of the patient. Thus, both conventional and smooth SMC algorithms compromise the safety and well-being of the patient under consideration.

The original PPO algorithm involves a nested loop that utilizes numerous episodes (the outer loop) that fix values of constants, which are employed by the algorithm for calculations of control action. And afterwards, the algorithm runs for specified number of iterations (inner loop) to work out the control actions using those particular fixed valued constants. The algorithm then updates these constants in the next episode. Eventually, running the algorithm for a large number of episodes will end up giving the best possible values of constants (or actor and critic weights) that provides the best possible results. Hence, the computational complexity of the original PPO algorithm is $O(n^2)$, as it involves a nested loop that runs for a specific number of episodes and implements the algorithm for a specific number of iterations within each episode^47,48^. However, this study simplifies the PPO algorithm by considering only one episode and minimizing the objective function by reiterating the algorithm, thus simplifying the computational complexity of the algorithm to *O*(*n*)^47,49^. Moreover, as RL-based algorithms do not utilize the mathematical model for control input calculations, thus both original PPO as well as proposed SSMC-PPO algorithms are model independent.

The proposed SSMC-PPO algorithm has depicted unique results shown in “Results”, as it not only mitigated tumor cell population in minimal time, but it also kept the administered dosages of radiation and chemotherapy at minimum. Moreover, after decimating tumorous cells, the control inputs successfully converge to zero. Similarly, the proposed algorithm administered the control inputs smoothly, that resulted in smooth dynamics of the system states. Thus, the overall toxicity of treatment procedure is minimal if proposed SSMC-PPO algorithm is employed for tumor mitigation using combined radiation and chemotherapy. The proposed algorithm is able to achieve these results as it employed an optimization-based approach to target multiple objectives simultaneously by intelligently adjusting the administered dosages of radiation and chemotherapeutic drug. Hence, the proposed SSMC-PPO algorithm clearly outperforms the remaining controllers, as it not only includes the swiftness and robustness of SMC and smoothness of sigmoid-based control, but also incorporates the optimizing capabilities of PPO.

### Statistical analysis

To further validate the performance of proposed sigmoid-SMC based PPO algorithm, the simulation based experiments performed in “Results” have been repeated multiple times by slightly modifying the initial conditions to statistically validate its performance using Mann-Whitney U test. The Mann-Whitney U test is used to provide non-parametric numerical evidence for three simulation based hypothesis: that the proposed algorithm administers lower radiation dosage, lower chemotherapeutic drug dosage, and a shorter treatment duration under the stated model assumptions. These tests support comparative simulation robustness, but they should not be interpreted as clinical statistical validation. For the first two hypothesis, the overall dosage administered by the proposed SSMC-PPO algorithm is compared to those administered by nonlinear SMC, smooth SMC and PPO algorithm, throughout the treatment procedure. Whereas, for the third hypothesis, recently proposed algorithms were also compared along with SMC, smooth SMC and PPO algorithms to statistically validate that the proposed SSMC-PPO algorithm effectively optimizes the administered treatment dosages while efficiently decimating tumorous mass in minimum time.

Thus, to statistically analyze and validate each hypothesis, repeated numerical solutions for each algorithm have been performed. For each considered algorithm, the model was simulated independently for n = 32 runs using identical parameter sets while varying the initial conditions as mentioned above. Each simulation run was treated as an independent numerical observation for the purpose of non-parametric comparison; however, these observations represent simulated model responses rather than independent clinical patients. The combined number of observations for all the three controllers for first two hypothesis is 32 + 32 + 32 = 96 independent observations. For the third hypothesis, a total number of 51 independent observations were recorded. Since, the resulting outcomes did not satisfy the assumptions of normality, as assessed by exploratory data analysis, a non-parametric statistical approach, the Mann-Whitney U test is adopted to statistically assess significant differences between pairs of considered groups. Similarly, all conducted statistical tests are two-sided, and a p-value of less than 0.05 is considered statistically significant. Moreover, all the statistical analyses are conducted using MATLAB.

#### Mann-Whitney U test—reduction in overall radiation

Three independent Mann-Whitney U tests have been performed to statistically evaluate the performance of proposed SSMC-PPO algorithm in comparison to nonlinear SMC, SSMC and RL based PPO algorithm. These tests are performed to statistically evaluate which of the considered algorithm administers minimum overall radiation dosage throughout the therapy. It is pertinent to mention here that these conducted experiments utilize multi-input approach, i.e. combined radio-chemotherapy to mitigate tumor cell population. However, only the administered radiation dosages, (by each considered algorithm) have been compared over here to investigate which algorithm cause least radiation related toxicity while successfully decimating tumor cell population. Identical initial conditions have been maintained during each individual experiment for all of the considered algorithms so that a more realistic and reliable analysis may be established. Each algorithm is tested 32 times by slightly varying the initial conditions and the attained data of the overall administered radiation dosage has been recorded in the four distinct groups. Each group represents the radiation dosages administered by the respective algorithm for different combinations of initial conditions. For instance, $group1_{\alpha }$ represents the radiation dosages administered by the proposed SSMC-PPO algorithm. A single element of the group represents the total radiation administered by the algorithm for a unique set of initial conditions. Similarly, the second element (and hence, the other remaining elements) represents total administered radiation dosage for slightly different but unique combination of initial conditions. $Group2_{\alpha }$ constitutes the radiation dosages administered by the PPO algorithm, whereas, $group3_{\alpha }$ and $group4_{\alpha }$ comprise the administered radiation dosage when SSMC and SMC were employed respectively. The recorded values of each group are given below:

$$group1_{\alpha }$$ = [13.81933, 13.78759, 13.78968, 13.77721, 13.76983, 13.77753, 13.75152, 13.79201, 13.81595, 13.81585, 13.7799, 13.78609, 13.79838, 13.80837, 13.80288, 13.78622, 13.78158, 13.78264, 13.7921, 13.77763, 13.7736, 13.79686, 13.80551, 13.77119, 13.75222, 13.78882, 13.7573, 13.76219, 13.75299, 13.77836, 13.76203, 13.79174]; *α*: SSMC-PPO

$$group2_{\alpha }$$ = [33.59543, 33.59544, 33.59544, 33.59544, 33.59544, 33.59544, 33.59544, 33.59544, 33.59545, 33.59545, 33.59545, 33.59545, 33.59545, 33.59545, 33.59546, 33.59546, 33.59546, 33.59546, 33.59546, 33.59546, 33.59546, 33.59547, 33.59547, 33.59547, 33.59547, 33.59547, 33.59547, 33.59547, 33.59548, 33.59548, 33.59548, 33.59548]; *α*: PPO

$$group3_{\alpha }$$ = [20.7015, 20.698, 20.6944, 20.6908, 20.6872, 20.6836, 20.68, 20.6765, 20.6729, 20.6693, 20.6657, 20.6622, 20.6586, 20.655, 20.6515, 20.6479, 20.6443, 20.6408, 20.6372, 20.6336, 20.6301, 20.6265, 20.623, 20.6194, 20.6159, 20.6123, 20.6087, 20.6052, 20.6016, 20.5981, 20.5945, 20.591]; *α*: SSMC

$$group4_{\alpha }$$ = [14.5697, 14.5698, 14.56985, 14.5699, 14.5699, 14.56997, 14.49, 14.4903, 14.8455, 14.8455, 14.70546, 14.7055, 14.70554, 14.70558, 14.70562, 14.76, 14.7632, 14.76322, 14.9405, 14.97824, 14.9783, 15.0544, 15.0544, 15.0545, 15.0545, 15.0545, 14.977, 14.977, 14.9772, 14.8859, 14.8859, 14.886]; *α*: SMC

The first test evaluates the performance of $group1_{\alpha }$ (SSMC-PPO) against $group2_{\alpha }$ (PPO). The second test evaluates $group1_{\alpha }$ (SSMC-PPO) against $group3_{\alpha }$ (SSMC), whereas, the last test compares the radiation dosages of $group1_{\alpha }$ (SSMC-PPO) with $group4_{\alpha }$ (SMC). For each of these tests, the following null hypothesis is considered:

Null hypothesis: There is no significant difference, in the overall radiation dosages, administered for tumor treatment, between the two considered groups.

Results: after performing the analysis, following results were obtained:

Test results: SSMC-PPO vs PPO

1. Hypothesis test result (h) = 1 : thus, null hypothesis is rejected
2. p-value = $5.787\times 10^{-12} < 0.05$ : as, the p-value is less than 0.05, it implies that null hypothesis is rejected and the observed results are very unlikely to occur by chance.
3. zval = -6.8848 : which indicates that $group1_{\alpha }$ or radiation dosages administered by proposed SSMC-PPO algorithm is significantly less toxic than that administered by PPO algorithm.
4. Rank sum = 528: which suggests that the radiation dosages for $group1_{\alpha }$ (SSMC-PPO) remain significantly and consistently lower than the dosages administered by $group2_{\alpha }$ (PPO)

Test results: SSMC-PPO vs SSMC

1. Hypothesis Test Result (h) = 1 : implying that the null hypothesis is rejected
2. p-value = $6.5113\times 10^{-12} < 0.05$ : indicating that the null hypothesis is rejected and there is statistically significant difference between the results of the two groups.
3. zval = -6.868 : verifying that the administered radiation dosages given in $group1_{\alpha }$ (by proposed SSMC-PPO algorithm) are significantly less toxic than that administered by SSMC algorithm given in $group3_{\alpha }$.
4. Rank sum = 528: suggesting that the radiation dosages for $group1_{\alpha }$ (SSMC-PPO) remain significantly and consistently lower than the dosages administered by $group3_{\alpha }$ (SSMC)

Test results: SSMC-PPO vs SMC

1. Hypothesis test result (h) = 1 : thus, null hypothesis is rejected
2. p-value = $6.4827\times 10^{-12} < 0.05$ :indicating that the null hypothesis is rejected and there is statistically significant difference between the results of the two considered groups.
3. zval = -6.8686 : this negative once again indicates that the radiation dosages administered by proposed SSMC-PPO algorithm in $group1_{\alpha }$ are significantly less toxic in comparison to those mentioned in $group4_{\alpha }$, administered by SMC.
4. Rank sum = 528: implying that proposed SSMC-PPO algorithm consistently administers significantly lesser radiation dosages in comparison to those administered by SMC.

**Table 5:** Statistical analysis: reduction in overall radiation.

Comparison	$n_1$	$Med_1$	$IQR_1$	$n_2$	$Med_2$	$IQR_2$	U-Stat.	z-value	p-value	r-value
$n_1$ vs $n_2$										
SSMC-PPO vs PPO	32	13.78	0.022	32	33.59	$2.5\times 10^{-5}$	0	-6.8848	$5.787\times 10^{-12}$	0.8606
SSMC-PPO vs SSMC	32	13.78	0.022	32	20.64	0.057	0	-6.868	$6.5113\times 10^{-12}$	0.8585
SSMC-PPO vs SMC	32	13.78	0.022	32	14.8	0.34	0	-6.8686	$6.4827\times 10^{-12}$	0.8586

$n_1$ number of observations of algorithm No. 1, $n_2$ number of observations of algorithm No. 2, *Med* median, *IQR* inter-quartile range, *U-Stat*. U-Statistics, *r-value* effect size, p-value significance threshold: 0.05. *SSMC-PPO* sigmoid sliding mode control-based proximal policy optimization, *PPO* proximal policy optimization, *SSMC* sigmoid function-based sliding mode control, *SMC* sliding mode control.

Significance: the results of the Mann-Whitney U-test are further elaborated and presented in Table 5. The results obtained after performing statistical analysis confirms that the administered radiation dosages by proposed SSMC-PPO algorithm are less intensive and thus result in decreased levels of toxicity due to radiation in comparison to the other considered algorithms.

#### Mann-Whitney U test—reduction in overall chemo-dosage

Radiation therapy is a targeted and directed procedure that concentrates high energy radiation on the tumorous tissue, thus the toxic impact of radiation remains relatively contained in comparison to the toxicity spread of chemotherapy. On the other hand, if the tumor is spreading expeditiously or is malignant in nature, then chemotherapy has to be applied in combination with radiation as discussed earlier in “Introduction”. As chemotherapeutic drug flows through blood stream to all the tumor sites, the therapy is hence far more toxic than radiotherapy. Thus, the algorithm that administers minimum chemotherapeutic dosage while effectively mitigating tumor cell population will result in least toxicity spread. To statistically analyze how much overall dosage is administered by the proposed SSMC-PPO algorithm in comparison to other considered techniques, three Mann-Whitney U tests have been performed. Again, as mentioned in “Mann-Whitney U test—reduction in overall radiation”, combined radio-chemotherapy has been administered but only chemotherapeutic drug administration by each algorithm is considered in these tests. Similarly, identical initial conditions have been maintained for all three algorithms to conduct these independent tests. Each algorithm is tested 32 times by slightly varying the initial conditions and the attained data of the overall administered chemotherapeutic drug dosage has been recorded in the four distinct groups. Each group value represents the chemotherapeutic drug dosage administered by the respective algorithm for a particular set of initial condition. Thus, $group1_{q}$ represents the chemotherapeutic drug dosages administered by the proposed SSMC-PPO algorithm. $Group2_{q}$ constitutes the chemo drug dosages administered by the PPO algorithm, whereas, $group3_{\alpha }$ and $group4_{\alpha }$ comprises the administered drug dosages when SSMC and SMC were employed respectively. The recorded values of each group are given below:

$$group1_{q}$$ = [13.46785, 13.47673, 13.47265, 13.46375, 13.46443, 13.47469, 13.47101, 13.47909, 13.4631, 13.47272, 13.49584, 13.48417, 13.45866, 13.46267, 13.48054, 13.4847, 13.49341, 13.46412, 13.4866, 13.49892, 13.46288, 13.48191, 13.48036, 13.48222, 13.47045, 13.48503, 13.50193, 13.49207, 13.47888, 13.49352, 13.46043, 13.46134]; q: SSMC-PPO

$$group2_{q}$$ = [33.59543, 33.59544, 33.59544, 33.59544, 33.59544, 33.59544, 33.59544, 33.59544, 33.59545, 33.59545, 33.59545, 33.59545, 33.59545, 33.59545, 33.59546, 33.59546, 33.59546, 33.59546, 33.59546, 33.59546, 33.59546, 33.59547, 33.59547, 33.59547, 33.59547, 33.59547, 33.59547, 33.59547, 33.59548, 33.59548, 33.59548, 33.59548]; q: PPO

$$group3_{q}$$ = [19.2533, 19.2478, 19.2423, 19.2368, 19.2314, 19.2259, 19.2205, 19.2152, 19.2098, 19.2045, 19.1993, 19.194, 19.1888, 19.1836, 19.1784, 19.1733, 19.1681, 19.1631, 19.158, 19.153, 19.148, 19.143, 19.1381, 19.1332, 19.1283, 19.1235, 19.1187, 19.1139, 19.1092, 19.1045, 19.0998, 19.0952]; q: SSMC

$$group4_{q}$$ = [46.3555, 46.356, 46.3565, 46.357, 46.35751, 46.358, 46.1285, 46.129, 45.6095, 45.61, 44.3405, 44.341, 44.3415, 44.342, 44.3425, 45.608, 45.6085, 45.609, 44.6745, 46.025, 46.0255, 46.346, 46.3465, 46.347, 46.3475, 46.348, 45.9386, 45.939, 45.9396, 47.33, 47.3306, 47.3311]; q: SMC

The first test evaluates the performance of $group1_{q}$ (SSMC-PPO) against $group2_{q}$ (PPO). The second test evaluates $group1_{q}$ (SSMC-PPO) against $group3_{q}$ (SSMC), whereas, the last test compares the chemotherapeutic drug dosages of $group1_{q}$ (SSMC-PPO) with $group4_{q}$ (SMC). For each of these tests, the following null hypothesis is considered:

Null hypothesis: There is no significant difference, in the overall chemotherapeutic drug dosages, administered for tumor treatment, between the two considered groups.

Results: After performing the analysis, following results were obtained:

Test results: SSMC-PPO vs PPO

1. Hypothesis Test Result (h) = 1 : thus, null hypothesis is rejected
2. p-value = $5.787\times 10^{-12} < 0.05$ : implying that the null hypothesis is rejected and difference between the dosages given in the two groups is statistically different.
3. zval = -6.8848 : which indicates that $group1_{q}$ or chemotherapeutic drug dosages administered by proposed SSMC-PPO algorithm is significantly lower, and hence less toxic than that administered by PPO algorithm.
4. Rank sum = 528: which suggests that the chemotherapeutic drug dosages given in $group1_{q}$ (SSMC-PPO) remain significantly and consistently lower than the dosages administered by $group2_{q}$ (PPO)

Test results: SSMC-PPO vs SSMC

1. Hypothesis Test Result (h) = 1 : implying that the null hypothesis is rejected
2. p-value = $6.5113\times 10^{-12} < 0.05$ : implying that the null hypothesis is rejected and difference between the dosages given in the two groups is statistically different.
3. zval = -6.868 : implying that $group1_{q}$ or chemotherapeutic drug dosages administered by proposed SSMC-PPO algorithm is significantly lower, and hence less toxic than that administered by SSMC algorithm.
4. Rank sum = 528: which suggests that the chemotherapeutic drug dosages given in $group1_{q}$ (SSMC-PPO) remain significantly and consistently lower than the dosages administered by $group3_{q}$ (SSMC).

Test results: SSMC-PPO vs SMC

1. Hypothesis Test Result (h) = 1 : thus, null hypothesis is rejected
2. p-value = $6.5113\times 10^{-12} < 0.05$ : implying that the null hypothesis is rejected and difference between the dosages given in the two groups is statistically different.
3. zval = -6.868 : this negative once again indicates that the chemotherapeutic drug dosages administered by proposed SSMC-PPO algorithm in $group1_{q}$ are significantly less toxic in comparison to those mentioned in $group4_{q}$, administered by SMC.
4. Rank sum = 528: which suggests that the chemotherapeutic drug dosages given in $group1_{q}$ (SSMC-PPO) remain significantly and consistently lower than the dosages administered by $group4_{q}$ (SMC).

**Table 6:** Statistical analysis: reduction in overall chemo dosage.

Comparison	$n_1$	$Med_1$	$IQR_1$	$n_2$	$Med_2$	$IQR_2$	U-Stat.	z-value	p-value	r-value
$n_1$ vs $n_2$										
SSMC-PPO vs PPO	32	13.47	0.02	32	33.5955	$2.5\times 10^{-5}$	0	-6.8848	$5.787\times 10^{-12}$	0.8606
SSMC-PPO vs SSMC	32	13.47	0.02	32	19.17	0.082	0	-6.868	$6.5113\times 10^{-12}$	0.8585
SSMC-PPO vs SMC	32	13.47	0.02	32	47.07	0.747	0	-6.868	$6.5113\times 10^{-12}$	0.8585

$n_1$ number of observations of algorithm No. 1, $n_2$ number of observations of algorithm No. 2, *Med* median, *IQR* inter-quartile range, *U-Stat.* U-statistics, *r-value* effect size, p-value significance threshold: 0.05. *SSMC-PPO* sigmoid sliding mode control-based proximal policy optimization, *PPO* proximal policy optimization, *SSMC* sigmoid function-based sliding mode control, *SMC* sliding mode control.

Significance: the results of the Mann–Whitney U-test are further elaborated and presented in Table 6. The obtained results once again proved that the proposed SSMC-PPO algorithm consistently administered lower dosages of chemotherapeutic drug, implying that the chemo dosage related toxicity also remains the lowest when compared to other three considered benchmarks.

#### Mann–Whitney U test—reduction in therapy time

The proposed SSMC-PPO algorithm utilizes both chemotherapy and radiation therapy simultaneously to eliminate the tumorous mass from the body. One of the prime objectives of utilizing this combined therapy is to make the anti-tumor procedure more potent against tumor proliferation so that patient outcomes may be improved significantly. The previous statistical analysis provided in “Mann-Whitney U test—reduction in overall radiation” and ”Statistical analysis” validates that the proposed SSMC-PPO algorithm is least toxic among the considered algorithms as it administers the least amount of drug and radiation dosages for successful tumor mitigation. However, minimizing treatment dosages without reducing overall therapy duration is undesirable as prolonged treatment not only results in resistance development by the tumor against chemo drugs but it also leads to a weakened immune system and ravaged health (due to treatment related toxicity). Thus, it is pertinent to mitigate tumorous mass as fast as possible to conserve health and improve patient outcomes. The statistical analysis provided in  “Mann-Whitney U test—reduction in overall radiation” and “Statistical analysis” statistically compares the performance of three algorithms with the proposed SSMC-PPO under identical system model, uniform parameter values, and identical initial conditions. In contrast, algorithms proposed in recent scientific studies are implemented on different mathematical models of disease dynamics having varied state definitions, parameter values, or structural complexity, making direct comparisons potentially misleading or inconclusive. However, to evaluate reduction in treatment duration by the proposed SSMC-PPO, algorithms provided in recent scientific articles, that explicitly quantify the treatment times, have been utilized. These recently proposed algorithms along with their treatment duration have been mentioned in Table 7.

**Table 7:** Statistics for hybrid SSMC-PPO and recently proposed algorithms.

Source	Baseline dosage		Tumor	Treatment	
			Mitigation	Intensity	
	Chemo (mg)	Rad (Gy)	Time (days)	Chemo (mg)	Rad (Gy)
Optimal-multi-input^10^	1	1	12	199	199
SMC^50^	0.45	–	15	66.2	–
Synergetic^2^	1	–	60	24	–
Statefeedback^2,50^	1	–	68	14.86	–
Fuzzy^2,28^	1	–	70	27.2	–
PID^2^	1	–	71	24.7	–
Optimal^20,23^	0.95	0.37	20	113	173
DRL TD3^7^	3.6	–	30	19.73	–
Supertwisting^29^	1	–	15	21.8	–
SSMC-PPO (Proposed)	0.3426	0.2312	7	13.4	13.76

*SMC* sliding mode control, *PID* proportional integral derivative, *DRL TD3* deep reinforcement learning-based twin delayed deep deterministic algorithm, *SSMC-PPO* sigmoid sliding mode control-based proximal policy optimization.

Thus, once again, Mann-Whitney U test is performed to statistically compare the treatment duration recorded (in days) while employing proposed SSMC-PPO algorithm against the techniques proposed in recent literature. The data is recorded in two separate groups. The values of group1 represent the treatment duration recorded when proposed multi-input SSMC-PPO has been utilized for tumor mitigation. Whereas, the values mentioned in group2 represent treatment duration for successful mitigation of tumorous mass when algorithms mentioned in Table 7 have been employed. As most of the recently proposed algorithms employ only chemotherapy for tumor eradication, thus, the treatment duration recorded by employing multi-input SMC, smooth SMC and PPO algorithms, provided in this research study, have also been included in group2 (last nine values). The recorded values of each group are given below:

*group*1 = [7, 7, 8, 8, 8, 9, 9, 9, 9, 9, 9, 9, 10, 10, 10, 10, 10, 10, 10, 9, 9, 9, 9, 9, 9, 8, 8, 8, 8, 8, 7]; (proposed SSMC-PPO)

*group*2 = [30, 50, 100, 200, 60, 68, 70, 100, 20, 30, 15, 22, 20, 25, 45, 45, 47, 3, 5, 6]; (Recently proposed algorithms in literature)

The following null hypothesis is considered for this test:

Null hypothesis: There is no significant difference, in the overall time duration of successful tumor treatment, between the two considered groups.

Results: After performing the analysis, following results were obtained:

Test results: SSMC-PPO vs Recently Proposed Algorithms in Literature

1. Total number of observations: 51
2. Hypothesis Test Result (h) = 1 : thus, null hypothesis is rejected
3. p-value = $2.3776\times 10^{-5} < 0.05$ : implying that the null hypothesis is rejected and difference between the time duration mentioned in the two groups is statistically different.
4. zval = -4.2261 : indicating that *group*1 or treatment duration observed when proposed SSMC-PPO algorithm is employed, is significantly lower, than the treatment periods mentioned in *group*2.
5. Rank sum = 589: suggesting that the duration given in *group*1 (SSMC-PPO) remain significantly and consistently lower than the duration mentioned in *group*2.
6. U-Statistic = 93: This signifies that some of the observations from the second group are better, i.e. they mitigate tumor cell population in lesser duration. However, most of the observations from the first group (SSMC-PPO) depict that the algorithm performed consistently to mitigate tumor cell population in lesser time.
7. Effect Size: 0.5918, signifies strong but not absolute dominance of the results of first group (SSMC-PPO) over second (recently proposed algorithms in literature).

The above-mentioned results of the Mann-Whitney U-test are further elaborated and presented in Table (8).

**Table 8:** Statistical analysis: reduction in overall therapy time.

Comparison	$n_1$	$Med_1$	$IQR_1$	$n_2$	$Med_2$	$IQR_2$	U-Stat.	z-value	p-value	r-value
$n_1$ vs $n_2$										
SSMC-PPO vs RPA	31	9	1	20	37.5	44	93	-4.2261	$2.3776\times 10^{-5}$	0.5918

$n_1$ number of observations of algorithm No. 1, $n_2$ number of observations of algorithm No. 2, *Med* median, *IQR* inter-quartile range, *U-Stat*. U-statistics, *r-value* effect size, p-value significance threshold: 0.05. *SSMC-PPO* sigmoid sliding mode control-based proximal policy optimization, *RPA* recently proposed algorithms in literature.

Thus, the results validate that the proposed sigmoid SMC-based PPO algorithm significantly reduces the time of convergence to bring tumor cell population to zero in comparison to the other recently proposed algorithms (mentioned in Table 7).

## Discussion: proposed sigmoid SMC-based PPO vs. algorithms proposed in recent studies

To further validate the superior performance of the proposed multi-input and multi-objective sigmoid-SMC based PPO algorithm, a detailed qualitative and quantitative comparison is presented in this section with the recently proposed anti-tumor algorithms in literature.

### Qualitative comparative analysis

In this section, the performance of the proposed sigmoid SMC-based PPO algorithm is compared qualitatively with those proposed in recent literature. The detailed comparison between them is provided in Table 9. The controllers and algorithms are compared on the basis of smoothness of the system response as well as the formulated control inputs by the algorithm, the mathematical model-dependency of the algorithm, mentioned as M-D, the type of therapy employed by the study and the metronomic flexibility of the algorithms mentioned as M-F in Table 9. Similarly, none of the recent studies considered the impact of the treatment therapies on human health and well-being. However, based on the drug dosages controlled by the respective algorithms; this negative impact on health is also considered as a comparison parameter. Similarly, the convergence of control inputs to 0 after successful tumor mitigation, and the time taken by the controller to do so, are also considered as performance comparison parameters. Wu et al.^22^ considered a switch system optimal control-based approach to manage the chemotherapeutic drug dosages for tumor reduction. Although the controller maintained low values of drug input, the algorithm was sluggish enough to cause an overshoot of 20% in the tumor cell population. A similar type of response was also observed in Dhanalakshmi et al.^28^, where the finite-time fuzzy controller resulted in an overshoot in the tumor cell population.

**Table 9:** Algorithm performance—qualitative comparison.

Source	Smooth response	M-D	Therapy type	M-F	Negative impact on	Input conv.	ST 2% (days)
Optimal control^22^	*×*	Yes	Chemo	*\checkmark*	Low	*\checkmark*	30
Optimal control^20^	*×*	Yes	Chemo	*\checkmark*	EH	*×*	50
AFBS-SMC^24^	*\checkmark*	Yes	N*\backslash*A	*×*	N *\backslash*A	*\checkmark*	100
Optimal control^10^	*×*	Yes	Chemo+Radio	*×*	EH	*\checkmark*	200
Fuzzy^2^a	*×*	Yes	Chemo	*×*	High	*\checkmark*	60
Synergetic^2^b	*×*	Yes	Chemo	*×*	High	*\checkmark*	68
State feedback^2^c	*×*	Yes	Chemo	*\checkmark*	High	*\checkmark*	70
Metronomic^39^	*×*	Yes	Chemo	*\checkmark*	Low	*\checkmark*	100
Optimal control^23^	*×*	Yes	Chemo	*×*	High	*×*	20
DRL^7^	*×*	No	Chemo	*\checkmark*	High	*\checkmark*	30
SST^29^	*\checkmark*	Yes	Chemo	*×*	VH	*×*	15
SSMC-PPO	*\checkmark*	No	Chemo+Radio	*\checkmark*	VL	*\checkmark*	8

*M-D* model dependency, *M-F* metronomic flexibility, *Conv*. convergence, *ST* settling time, *EH* extremely high, *N**\backslash**A* not available, *VH* very high, *VL* very low, *AFBS-SMC* adaptive fuzzy backstepping-based sliding mode control, *DRL* deep reinforcement learning, *SST* smooth supertwisting, *SSMC-PPO* sigmoid sliding mode control-based proximal policy optimization, *Chemo* chemotherapy, *Radio* radiation therapy.

Shindi et al.^20^ exploited PSO with an optimal controller to develop a chemotherapy-based treatment plan. Not only is the developed controller computationally very costly, but it also suggests extremely high drug dosages (mentioned as EH in Table 9), making it inappropriate because of patient’s safety concerns. Sarhaddi et al.^24^ devised backstepping and SMC-based adaptive fuzzy controller. However, the study did not specify the type of therapy utilized by the controller to eradicate the tumor population. Moreover, the inputs generated by the controller also become negative, which is practically impossible, thus limiting the scope and practicality of the devised method. Kumar et al.^10^ considered combining radio and chemotherapy for tumor treatment by employing an optimal controller. The algorithm however, devised such a treatment plan that suggests extremely high levels of radiation and drug dosages for a long duration of 200 days. Even if the tumor population effectively becomes zero swiftly, the controller still pumps maximum dosages of control input to the system, making the controller highly inefficient for such sensitive applications of radiation control and drug regulation.

Qaiser et al.^2^ suggested nonlinear synergetic (a), state feedback (b) and fuzzy logic-based controllers (c) for tumor reduction. Whereas, Arora et al.^39^ employed a metronomic scheduling technique for the chemotherapy of tumor cells. Both Qaiser et al.^2^ and Arora et al.^39^ proposed controllers that took quite a long time to bring the tumor population to 0. Moreover, their devised control actions did not consider transients or their adverse effects on patient’s well-being. Sena et al.^23^ also suggested an optimal fuzzy logic-based $P+D^\mu$ controller to adjust chemotherapeutic drug dosages for cancer treatment. Like Qaiser et al.^2^, the fuzzy controller proposed by Sena et al.^23^ also resulted in high drug dosages with fairly high transients in control input as well as tumor dynamics. Mashayekhi et al.^7^ employed a computationally costly deep reinforcement learning TD3 algorithm. Although the algorithm suggested high drug dosages for tumor treatment, the algorithm is itself model independent, i.e. it does not require mathematical dynamical equations to determine the drug dosages. Moreover, the algorithm also adjusts the control input haphazardly, which introduces transients in the system dynamics and thus, it is inconvenient for practical use. Finally, Rsetam et al.^29^ utilized super-twisting algorithm to adjust the chemotherapy dosage for tumor treatment. The algorithm not only devised very high drug dosages (mentioned as VH in Table 9) for the therapy, but also resulted in negative chemotherapeutic drug dosages suggesting that the algorithm somehow removes the drug from the system, which is impossible. Thus, after detailed comparison, it can be inferred that the proposed novel sigmoid SMC-based PPO algorithm clearly outperforms the other recently proposed algorithms and controllers for the application of tumor reduction.

**Table 10:** Algorithm performance—quantitative comparison with proposed SSMC-PPO.

Source	Baseline dosage reduction		Faster tumor reduction	Treatment intensity reduction	
	Chemo	Radio		Chemo	Radio
Optimal-multi-input^10^	76.8%	66%	5.67%	92.45%	92.3%
SMC^50^	24%	–	53.33%	79.2%	–
Synergetic^2^	76.8%	–	88.33%	37%	–
Statefeedback^2,50^	76.8%	–	89.7%	1.7%	–
Fuzzy^2,28^	76.8%	–	90%	44%	–
PID^2^	76.8%	–	90%	39.6%	–
Optimal^20,23^	64.2%	37.56%	65%	87.8%	92.25%
DRL TD3^7^	91%	–	76.6%	24.12%	–
Supertwisting^29^	83.4%	–	53.3%	32.3%	–
Sig-SMC (“Conventional SMC vs sigmoid function-based smooth SMC”)	4.46%	2%	93%	25.8%	66.7%

*SMC* sliding mode control, *PID* proportional integral derivative, *DRL TD3* deep reinforcement learning-based twin delayed deep deterministic algorithm, *Sig-SMC-PPO* sigmoid function-based sliding mode control.

### Quantitative comparative analysis

Since the algorithms proposed in recent literature have been implemented and tested on various tumor-system models, that involve different initial conditions and treatment therapies, thus a more in-depth comparison has been offered to establish the superiority of the proposed sigmoid SMC-based PPO algorithm. The comparison provided in Table 10 assesses the performance of algorithms proposed in recent times, for tumor mitigation, on the basis of three parameters. The first parameter is the reduction in baseline or initial dosage done by the proposed sigmoid SMC-based PPO algorithm in comparison with the other recently proposed algorithms mentioned in Table 10. Similarly, the second parameter of comparison explains how much faster the proposed hybrid PPO algorithm is, in comparison to other techniques employed for tumor mitigation. Lastly, the third parameter in Table 10 illustrates the reduction in total chemo drug and radiation dosages over the span of whole combined therapy, administered by the proposed algorithm in comparison to the other recent techniques. For instance, the multi-input optimization based radio-chemotherapy presented in^10^, also proposed simultaneous action of two anti-tumor therapies to mitigate tumor cells more effectively. Yet, the optimization based multi-input controller (presented in^10^) computed a staggering 76.8% and 66% higher baseline chemo and radiation dosages in comparison to the proposed sigmoid SMC-based PPO algorithm. Similarly, the proposed sigmoid SMC-based PPO algorithm is not only 5.67% faster than the technique presented in^10^ but it also utilized 92.45% and 92.3% less chemotherapeutic drug and radiation dosages respectively, throughout the therapy. Under the stated simulation conditions and the reported comparison framework, the proposed hybrid algorithm demonstrates substantially lower treatment intensity and faster tumor suppression than the compared approaches from recent literature. Because these algorithms may differ in model structure, assumptions, and therapeutic settings, the comparison should be interpreted as evidence of strong computational performance, rather than direct clinical superiority. The main reason for high baseline dosages computed by the recently proposed approaches is their inability to consider and prioritize the health and safety of the patient under consideration. These algorithms generally tend to evaluate the control input dosages based on the error function only. Hence, by combining nonlinear sigmoid SMC with PPO, this study successfully exploited the fast convergence and robustness of nonlinear control with the RL based optimization of PPO, resulting in fastest tumor reduction, least treatment intensity and baseline dosages in comparison to recently proposed algorithms.

## Limitations and practical implications

Although the proposed algorithm demonstrates effectiveness and robustness in simulation studies, its present contribution should be interpreted as a pre-clinical computational proof of concept. Several significant challenges and limitations must be addressed before practical clinical translation:

- The ODE based framework adopted in this study is formulated to achieve a balance between practicality and tractability. Yet the model cannot fully encapsulate the highly nonlinear and multi-faceted nature of biological systems encountered in practical clinical settings. The implication of combined radio-chemotherapy is strongly governed by a number of patient-specific physiological conditions and underlying health factors. Moreover, tumors are characterized by heterogeneity, including variation in growth kinetics, regression patterns, and differing levels of treatment sensitivity and resistance. These considerations highlight the limitations of uniform treatment strategies and motivate the need for personalized therapeutic approaches capable of accurately modeling tumor-specific responses and optimizing intervention strategies accordingly.
- Accurate and timely acquisition of system state information and therapeutic input levels are necessary to effectively utilize the complete potential of recently developed anti-tumor control strategies. in a practical clinical environment, exact measurements of all relevant system states are often subjected to uncertainty. Consequently, the deployment of such algorithms necessitates the integration of state estimation frameworks to address the uncertainties regarding state information.
- The translation of proposed control strategy into practical and clinical environment necessitates strict adherence to established safety and efficacy regulations, imposed by international and national governing bodies such as Food and Drug Administration (FDA) and European Medicines Agency (EMA). Furthermore, healthcare professionals would require specialized training to ensure proficient operation of the control framework. Such measures are essential to enable a controlled and safe transition from simulation-based investigation to real-world clinical deployment, while effectively mitigating potential risks associated with clinical application.
- It is pertinent to note that the proposed ODE-based control framework can be interpreted as a high-level decision-support mechanism and dosage regulation layer that complements emerging experimental and clinical treatment strategies. Within this context, the model and control strategy employed in this study serve as a control-oriented tool designed to assist in predicting and adjusting the therapeutic dosages in response to evolving dynamics of tumor progression.

## Future research directions

While the present research study demonstrates the effectiveness of the proposed SSMC-PPO framework for optimal chemotherapy and radiotherapy scheduling, several concerns for future research remain open. The considered system model can be extended to incorporate more detailed pharmaco-kinetic and pharmaco-dynamic descriptions of chemo and radiation effects. Additionally, future studies may consider alternative or additional health indicators beyond BMI to capture broader aspects of patient well-being. Similarly, to broaden the impact of this work, future research must focus on incorporating patient-specific adaptations into the underlying mathematical model through the integration of individualized clinical data. Finally, retrospective validation using clinical datasets, patient-specific parameterization, uncertainty quantification, and prospective safety evaluation represent essential steps before the proposed control methodology can be considered for practical decision-support use in personalized cancer therapy.

## Conclusion

This paper proposed a novel sigmoid SMC-based PPO algorithm to treat tumor progression. The study also updated a recent multi-input tumor-immune system dynamical model by incorporating the impact of harmful chemotherapeutic drugs and radiation on the general health and well-being of patients under consideration. The nonlinear system model has been utilized to initially design sigmoid function-based smooth SMC which is then incorporated within the framework of RL-based PPO algorithm. The stability analysis, based on Lyapunov stability theory was also presented to ensure the convergence of tumor cell population to zero. The performance of model-independent, multi-input sigmoid SMC-based PPO algorithm was analyzed through simulations via MATLAB. To validate the effectiveness of the designed controller, its performance was compared to those of SMC, sigmoid function-based smooth SMC and PPO algorithms, as well as with 11 recently proposed algorithms and controllers from literature. The observations and results are presented and the merits and demerits of each technique are discussed in detail. Although all the considered controllers successfully reduced the tumor cell population and inhibited tumor growth, the study revealed that the SMC, smooth SMC and PPO are not as beneficial for tumor reduction as the proposed SSMC-PPO algorithm. The proposed sigmoid SMC-based PPO method was not only found to be robust and efficient, but it also optimized the system inputs, resulting in a smooth response of system dynamics. Thus, the algorithm maintained very low values of control actions, while effectively decimating the tumor cells. Within the stated simulation framework, the administration of relatively lower dosages of radiation and chemotherapeutic drug by the proposed SSMC-PPO algorithm indicates reduced treatment-toxicity burden while maintaining tumor suppression. These findings support the methodological value and translational potential of the proposed toxicity-aware control framework, while further retrospective and prospective clinical validation is required before any claim of patient-level health benefit can be made.

## Data Availability

The datasets generated and analyzed during the current study are publicly available at: GitHub Repository for generated and analyzed data of the considered algorithms: Ref.^51^. The repository contains complete simulation-generated datasets in CSV format, including raw simulation outputs and processed datasets used for statistical analysis and performance evaluation for the considered and proposed control algorithms. The repository includes all scripts and instructions required to reproduce the simulation results, figures, and statistical analysis presented in this paper. Furthermore, the repository also includes a detailed description of the structure of recorded data. Whereas, the relationship between datasets, simulation code and reported results is also provided within the description file to ensure transparency and reproducibility of the study findings.

## Abbreviations

ODE: Ordinary differential equation
SMC: Sliding mode control
PPO: Proximal policy optimization
TMZ: Temozolomide
MRI: Magnetic resonance imaging
PSO: Particle swarm optimization
PD: Proportional-derivative
MPC: Model predictive control
RL: Reinforcement learning
ML: Machine learning
TD3: Twin delayed deep deterministic
SSMC-PPO: Sigmoid function-based sliding mode control-based proximal policy optimization
SSMC: Sigmoid function-based smooth sliding mode control
BMI: Body mass index
RT: Rise time
FT: Fall time
ST: Settling time
SSE: Steady state error
VH: Very high
EH: Extremely high
PID: Proportional integral derivative
Sig-SMC: Sigmoid function-based SMC
DRL: Deep reinforcement learning
M-D: Model dependence
M-F: Metronomic flexibility
EH: Extremely high
N\A: Not applicable
VH: Very high
VL: Very low
AFBS-SMC: Adaptive fuzzy backstepping-based sliding mode control
SST: Smooth super twisting
FDA: Food and Drug Administration
EMA: European Medicines Agency

## Code availability

The MATLAB codes used to generate the results of this study are publicly available at: (1) GitHub Repository for proposed sigmoid SMC-based PPO algorithm: Ref.^52^. (2) GitHub Repository for utilized PPO algorithm: Ref.^53^. (3) GitHub Repository for utilized SMC and smooth SMC algorithms: Ref.^54^. The repositories include all scripts and instructions required to reproduce the simulation results, figures and statistical analyses presented in this paper.

## References

[1] Tang, Y., Su, H., Jin, T. & Flesch, R. C. C. Adaptive PID control approach considering simulated annealing algorithm for thermal damage of brain tumor during magnetic hyperthermia. *IEEE Trans. Instrum. Meas.* **72**, 1–8. 10.1109/TIM.2023.3242011 (2023).37323850

[2] Qaiser, H., Ahmad, I. & Kashif, M. Fuzzy, synergetic and non-linear state feedback control of chemotherapy drug for a cancerous tumor. *Biomed. Signal Process. Control* **62**, 102061. 10.1016/j.bspc.2020.102061 (2020).

[3] American Cancer Society. Global Cancer Facts & Figures. https://www.cancer.org/research/cancer-facts-statistics/global-cancer-facts-and-figures.html (2024).

[4] American Cancer Society. Global Cancer Statistics. https://pressroom.cancer.org/GlobalCancerStatistics2024 (2024).

[5] American Cancer Society. Survival rates for bladder cancer. https://www.cancer.org/cancer/types/bladder-cancer/detection-diagnosis-staging/survival-rates.html (2024).

[6] Parisa, Y., Nader, M., Al-Naemi, Mostafa, A. & Levente, K. Reinforcement learning-based control of tumor growth under anti-angiogenic therapy. *Comput. Methods Prog. Biomed.* **173**, 15–26. 10.1016/j.cmpb.2019.03.004. (2019). 31046990

[7] Mashayekhi, H., Nazari, M., Jafarinejad, F. & Meskin, N. Deep reinforcement learning-based control of chemo-drug dose in cancer treatment. *Comput. Methods Prog. Biomed.* **243**, 107884. 10.1016/j.cmpb.2023.107884 (2024). 37948911

[8] Dixon, M., Phan, T. A., Dallon, J. & Tian, J. P. Mathematical model for IL-2-based cancer immunotherapy. *Math. Biosci.* **372**, 109187. 10.1016/j.mbs.2024.109187 (2024). 38575057 PMC11193449

[9] Gonzalez, I., Gomez, A., Pouso, O. L., Fenwick, J. D. & Pardo, J. A biomathematical model of tumor response to radioimmunotherapy with alpha-PDL1 and alpha-CTLA4. *IEEE/ACM Trans. Comput. Biol. Bioinform.* **20**, 808–821. 10.1109/TCBB.2022.3174454 (2023). 35544486

[10] Kumar, A., Dubey, U. S. & Dubey, B. The impact of radio-chemotherapy on tumour cells interaction with optimal control and sensitivity analysis. *Math. Biosci.* **369**, 109146. https://linkinghub.elsevier.com/retrieve/pii/S0025556424000063. (2024). 10.1016/j.mbs.2024.109146 38246323

[11] Gaiaschi, L., Bottone, M. G. & De Luca, F. Towards effective treatment of glioblastoma: The role of combination therapies and the potential of phytotherapy and micotherapy. *Curr. Issues Mol. Biol.* **46**, 14324–14350. https://www.mdpi.com/1467-3045/46/12/859. (2024). 10.3390/cimb46120859 PMC11674107 39727987

[12] Stupp, R. et al. Radiotherapy plus concomitant and adjuvant temozolomide for glioblastoma. *N. Engl. J. Med.* **352**, 987–996. 10.1056/NEJMoa043330 (2005). 15758009

[13] Angom, R. S., Nakka, N. M. R. & Bhattacharya, S. Advances in glioblastoma therapy: An update on current approaches. *Brain Sci.* **13**. 10.3390/brainsci13111536. (2023). PMC10669378 38002496

[14] Duwe, G. et al. Hepatotoxicity following systemic therapy for colorectal liver metastases and the impact of chemotherapy-associated liver injury on outcomes after curative liver resection. *Eur. J. Surg. Oncol.* **43**, 1668–1681. 10.1016/j.ejso.2017.05.008 (2017). 28599872

[15] Alghamdi, M. et al. Outcomes of patients with metastatic colorectal cancer treated with trifluridine/tipiracil beyond the second line: A multicenter retrospective study from Saudi Arabia. *J. Oncol.* **2022**, 3796783. 10.1155/2022/3796783 (2022). 36147443 PMC9485708

[16] Davis, A., Gao, R. & Navin, N. Tumor evolution: Linear, branching, neutral or punctuated? *Biochim. Biophys.Acta (BBA) - Rev. Cancer* **1867**, 151–161. 10.1016/j.bbcan.2017.01.003. (2017). PMC5558210 28110020

[17] Anna, P. et al. Combined mechanistic modeling and machine-learning approaches in systems biology – A systematic literature review. *Comput. Methods Prog. Biomed.* **240**, 107681. 10.1016/j.cmpb.2023.107681. (2023). 37385142

[18] Arsalan, M., Muhammad, S. & Sadiq, M. T. Nonlinear smooth sliding mode control framework for a tumor-immune dynamical system under combined radio-chemotherapy. *Mathematics* **14**. 10.3390/math14030521. (2026).

[19] Sharifi, N., Ozgoli, S. & Ramezani, A. Multiple model predictive control for optimal drug administration of mixed immunotherapy and chemotherapy of tumours. *Comput. Methods Prog. Biomed.* **144**, 13–19. 10.1016/j.cmpb.2017.03.012 (2017). 28494997

[20] Shindi, O., Kanesan, J., Kendall, G. & Ramanathan, A. The combined effect of optimal control and swarm intelligence on optimization of cancer chemotherapy. *Comput. Methods Prog. Biomed.* **189**, 105327. 10.1016/j.cmpb.2020.105327 (2020). 31978808

[21] Spyridon, P. et al. Tumor growth modeling. Parameter estimation with maximum likelihood methods. *Comput. Methods Prog. Biomed.* **160**, 1–10. 10.1016/j.cmpb.2018.03.014 (2018). 29728236

[22] Wu, X., Hou, Y. & Zhang, K. Switched system optimal control approach for drug administration in cancer chemotherapy. *Biomed. Signal Process. Control* **75**, 103575. 10.1016/j.bspc.2022.103575 (2022).

[23] Ay, S. & Soylu, S. Optimal fuzzy P + D controller for cancer chemotherapy. *Biomed. Signal Process. Control* **96**, 106634. 10.1016/j.bspc.2024.106634 (2024).

[24] Sarhaddi, M. & Yaghoobi, M. A new approach in cancer treatment regimen using adaptive fuzzy back-stepping sliding mode control and tumor-immunity fractional order model. *Biocybern. Biomed. Eng.* **40**, 1654–1665. 10.1016/j.bbe.2020.09.003 (2020).

[25] Ma, S. et al. Association of body mass index with outcomes among patients with head and neck cancer treated with chemoradiotherapy. *JAMA Netw. Open* **6**, e2320513–e2320513. 10.1001/jamanetworkopen.2023.20513 (2023). 37368400 PMC10300672

[26] American Cancer Society. Managing cancer-related side effects. https://www.cancer.org/cancer/managing-cancer/side-effects.html.

[27] Reşat, D. & Özgür. Angiogenic inhibition therapy, a sliding mode control adventure. *Comput. Methods Prog. Biomed.* **190**, 105358. 10.1016/j.cmpb.2020.105358 (2020). 32036204

[28] Dhanalakshmi, P., Senpagam, S. & Mohanapriya, R. Finite-time fuzzy reliable controller design for fractional-order tumor system under chemotherapy. *Fuzzy Sets Syst.* **432**, 168–181. 10.1016/j.fss.2021.06.013 (2022).

[29] Rsetam, K., Al-Rawi, M., Cao, Z., Alsadoon, A. & Wang, L. Model based smooth super-twisting control of cancer chemotherapy treatment. *Comput. Biol. Med.* **169**, 107957. 10.1016/j.compbiomed.2024.107957 (2024). 38190767

[30] Arsalan, M., Yu, X., Sadiq, M. T. & Almogren, A. Simultaneous multi-treatment strategy for brain tumor reduction via nonlinear control. *Brain Sci.* **15**. 10.3390/brainsci15020207. (2025). PMC11853036 40002539

[31] Zubair, M., Rana, I. A., Islam, Y. & Khan, S. A. Variable structure based control for the chemotherapy of brain tumor. *IEEE Access* **9**, 107333–107346. 10.1109/ACCESS.2021.3091632 (2021).

[32] Jiao, H., Shen, Q., Shi, Y. & Shi, P. Adaptive tracking control for uncertain cancer-tumor-immune systems. *IEEE/ACM Trans. Comput. Biol. Bioinform.* **18**, 2753–2758. 10.1109/TCBB.2020.3036069 (2021). 33156791

[33] Pariya, K. & Ramin, V. Optimal control design for drug delivery of immunotherapy in chemoimmunotherapy treatment. *Comput. Methods Prog. Biomed.* **229**, 107248. 10.1016/j.cmpb.2022.107248 (2023). 36463673

[34] Pantelis, S., Panagiotis, P. & Haralambos, S. Robust model predictive control for optimal continuous drug administration. *Comput. Methods Prog. Biomed.* **116**, 193–204. 10.1016/j.cmpb.2014.06.003 (2014). 24986530

[35] Mohite, U. L. & Patel, H. G. Optimization assisted Kalman filter for cancer chemotherapy dosage estimation. *Artif. Intell. Med.* **119**, 102152. 10.1016/j.artmed.2021.102152 (2021). 34531011

[36] Belkhatir, Z. et al. Stochastic Norton–Simon–Massagué tumor growth modeling: Controlled and mixed-effect uncontrolled analysis. *IEEE Trans. Control Syst. Technol.* **29**, 704–717. 10.1109/TCST.2020.2975141 (2021).

[37] Javadi, A., Keighobadi, F., Nekoukar, V. & Ebrahimi, M. Finite-set model predictive control of melanoma cancer treatment using signaling pathway inhibitor of cancer stem cell. *IEEE/ACM Trans. Comput. Biol. Bioinform.* **18**, 1504–1511. 10.1109/TCBB.2019.2940658 (2021). 31514151

[38] Villa-Tamayo, M. F., Anelone, A. J. N. & Rivadeneira, P. S. Tumor reduction using oncolytic viruses under an impulsive nonlinear estimation and predictive control scheme. *IEEE Control Syst. Lett.* **5**, 1705–1710. 10.1109/LCSYS.2020.3043185 (2021).

[39] Arora, G., Bairagi, N. & Chatterjee, S. A mathematical model to study low-dose metronomic scheduling for chemotherapy. *Math. Biosci.* **372**, 109186. 10.1016/j.mbs.2024.109186 (2024). 38580078

[40] Amir, Z., Ebrahimi & et al. Reinforcement learning for optimal scheduling of glioblastoma treatment with temozolomide. *Comput. Methods Prog. Biomed.* **193**, 105443. 10.1016/j.cmpb.2020.105443. (2020). 32311510

[41] Gino, J. L., Laleh, K., Saba, E. & Wenhua, C. A risk-based modeling approach for radiation therapy treatment planning under tumor shrinkage uncertainty. *Eur. J. Oper. Res.* **280**, 266–278. 10.1016/j.ejor.2019.06.041 (2020).

[42] Saba, E. & Gino, J. L. A reinforcement learning approach for finding optimal policy of adaptive radiation therapy considering uncertain tumor biological response. *Artif. Intell. Med.* **85**, 102193. 10.1016/j.artmed.2021.102193 (2021). 34763808

[43] Donghoon, L. et al. Deep learning driven predictive treatment planning for adaptive radiotherapy of lung cancer. *Radiother. Oncol.* **169**, 57–63. 10.1016/j.radonc.2022.02.013. (2022). PMC9018570 35189155

[44] Chan-Yun, Y. et al. Reinforcement learning strategies in cancer chemotherapy treatments: A review. *Comput. Methods Prog. Biomed.* **229**, 1072803. 10.1016/j.cmpb.2022.107280. (2023). 36529000

[45] Regina, P., Nader, M. & Wassim, M. H. Reinforcement learning-based control of drug dosing with applications to anesthesia and cancer therapy. *Control Appl. Biomed. Eng. Syst.* 251–297. 10.1016/B978-0-12-817461-6.00009-3. (2020).

[46] Chen, L., Zhang, Y.-W. & Zhang, S.-C. Optimal drug dosage control strategy of immune systems using reinforcement learning. *IEEE Access* **11**, 1269–1279. 10.1109/ACCESS.2022.3233567 (2023).

[47] Yang, G. et al. Proximal policy optimization with policy feedback. *IEEE Trans. Syst. Man Cybern. Syst.* **52**, 4600–4610. 10.1109/TSMC.2021.3098451 (2022).

[48] Yang, G. et al. Anti-martingale proximal policy optimization. *IEEE Trans. Cybern.* **53**, 6421–6432. 10.1109/TCYB.2022.3170355 (2023). 35560090

[49] Yuhu, C. et al. Proximal policy optimization with advantage reuse competition. *IEEE Trans. Cybern.* **5**, 3915–3925. 10.1109/TAI.2024.3354694 (2024).

[50] Zubair, M. et al. Lyapunov based nonlinear controllers for the chemotherapy of brain tumor. *Biomed. Signal Process. Control* **68**, 102804. 10.1016/j.bspc.2021.102804 (2021).

[51] Arsalan, M. Statistical analysis data. https://github.com/Arsalan-017/Data_Scientific_Reports_RL-based_Optimizer (2026).

[52] Arsalan, M. Matlab code for: Tumor mitigation using smooth smc-based ppo algorithm. https://github.com/Arsalan-017/Tumor_Mitigation_SSMC-PPO (2026).

[53] Arsalan, M. Matlab code for: Tumor mitigation using ppo algorithm. https://github.com/Arsalan-017/Tumor_Mitigation_PPO (2026).

[54] Arsalan, M. Matlab code for: Tumor mitigation using conventional smc and smooth smc algorithms. https://github.com/Arsalan-017/Tumor-Mitigation-using-SMC-and-Smooth-SMC (2026).

